# Supplementary material for: Nrf2 negatively regulates STING indicating a link between antiviral sensing and metabolic reprogramming
Source: Nat Commun. 2018 Aug 29;9:3506. doi: 10.1038/s41467-018-05861-7 (PMC6115435; doi:10.1038/s41467-018-05861-7)
Supplement: Supplementary file 1 — Supplementary Information [file 41467_2018_5861_MOESM1_ESM.pdf]

Supplementary Figures and Methods for:

Nrf2 negatively regulates STING indicating a link between antiviral sensing and metabolic reprogramming

Olagnier et al.,

Supplementary Figures

Olagnier et al.,

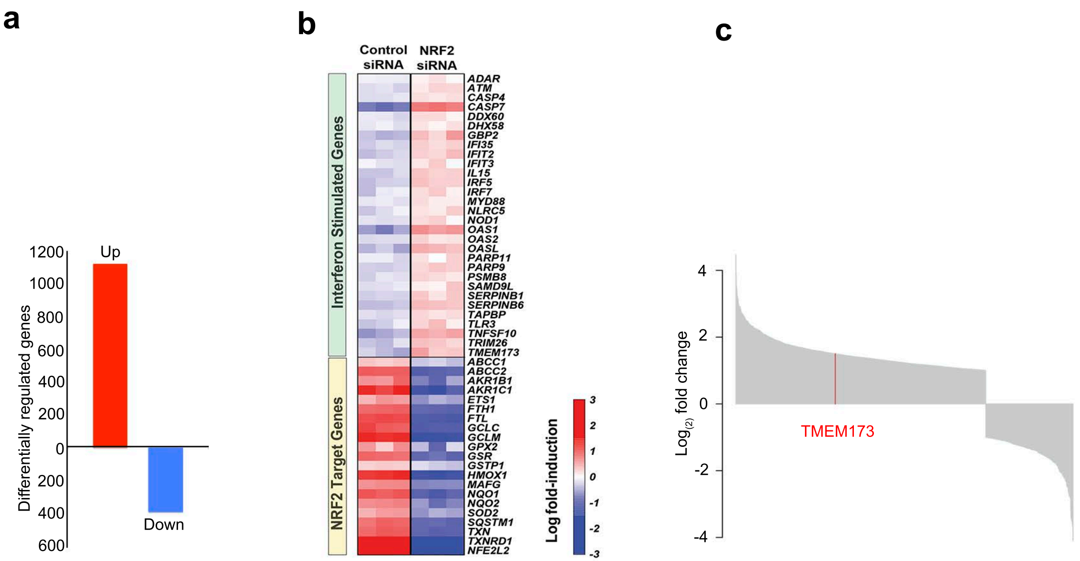

Supplementary Figure 1. RNA sequencing data from A549 cells silenced for Nrf2.

a) A graph depicting the number og total up- and down-regulated genes from the RNA seq analysis of si ctrl and si Nrf2-treated cells.

b) A heatmap specifically displaying fold-changes of Interferon Stimulated genes and known Nrf2 target genes.

c) Waterfall graphic showing the differentially regulated genes and the position of *TMEM173*.

Data are based on one RNAseq data set from one experiment perfromed in triplicate.

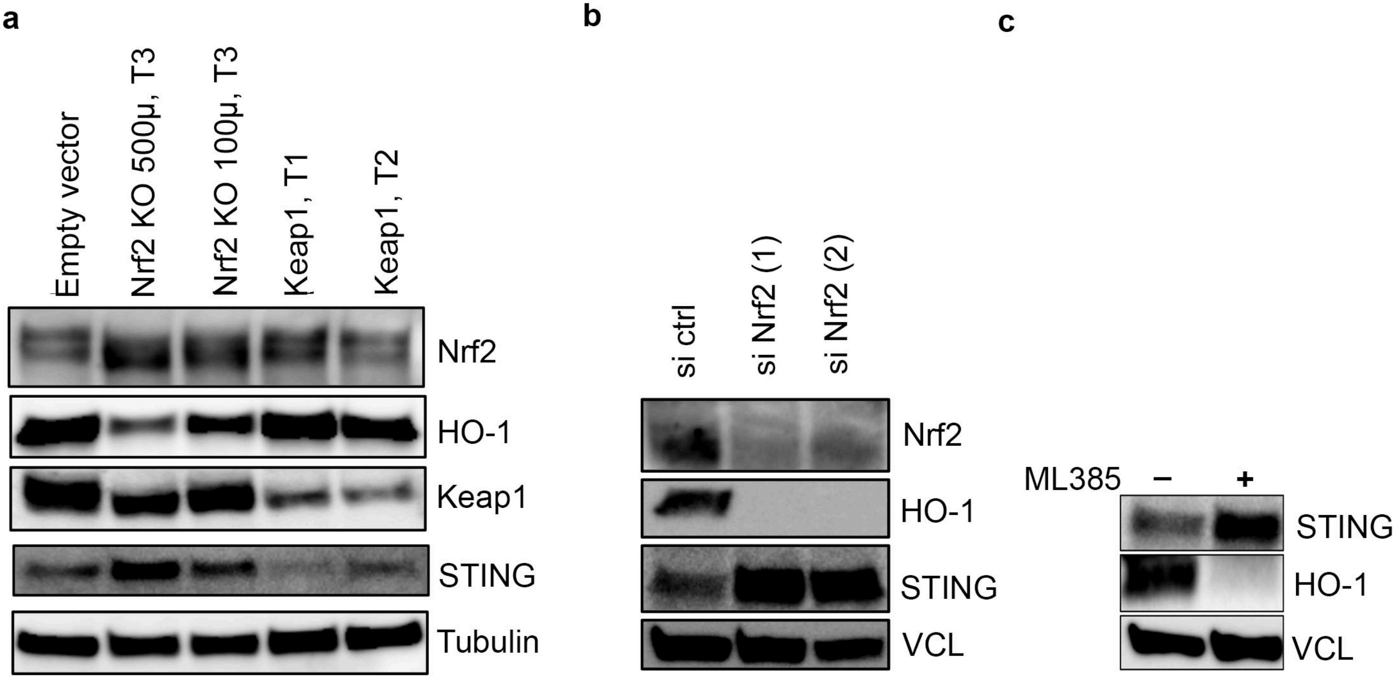

Supplementary Figure 2. Nrf2 suppresses STING expression.
a) A549 cells were treated with lentivirus carrying expression plasmids for Cas9 and specific RNA guides for either Nrf2 (one guide, different lentivirus dose) or Keap1 (two guides, T1 and T2). Cells were then selected by puromycin for two weeks. Samples were analysed by western blotting for Nrf2, HO-1, Keap1, STING and Tubulin as loading control. Blot displays data from one experiment.
b) A549 cells were treated with two different siRNA sequences both targeting Nrf2 or with control siRNA (si ctrl) for 48 hours. Cells were then lysed and samples were analysed by western blotting for Nrf2, HO-1,STING and Vinculin (VCL) as loading control. Blot displays data from one experiment.
c) A549 cells were treated with the inhibitor ML385, which blocks the ability of Nrf2 to bind DNA for 24 hours. Cells were then lysed and samples were analysed by western blotting for STING, HO-1, and Vinculin (VCL) as loading control.Blot is representative of two independent experiments.
See also Fig 1.

.

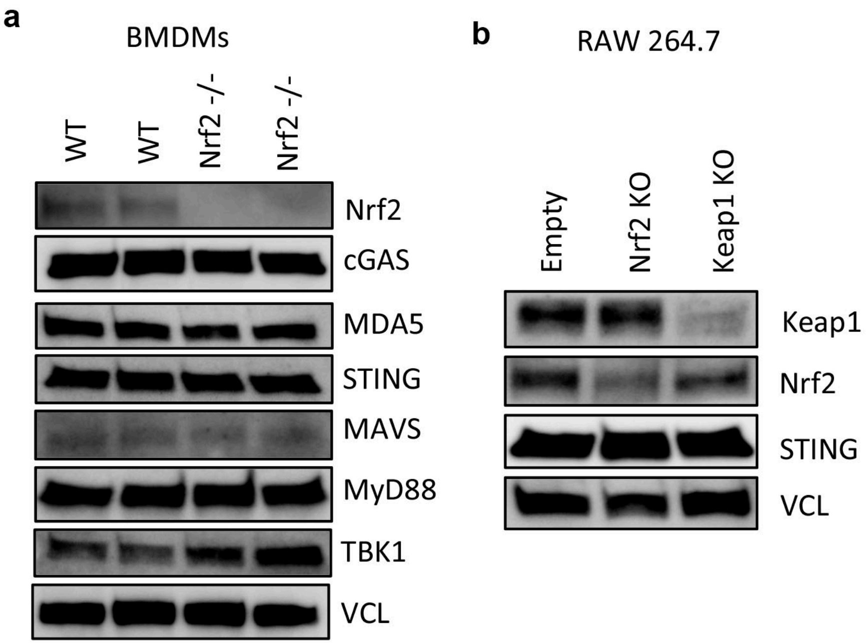

Supplementary Figure 3. Nrf2 does not suppress STING in murine BMDMs or RAW264.7 cells
a) Bone-marrow derived macrophages from wildtype (WT) or Nrf2 KO (Nrf2–/–) mice were analyzed by western blotting. Blot displays data from one experiment using two sets of mice.
b) Expression of Nrf2 or Keap1 was eliminated from Raw 264.7 cells using CrisprCas9 technology. After puromycin selection cells were analyzed by western blotting. Blot shows is representative of two independent experiments.
See also Fig 1.

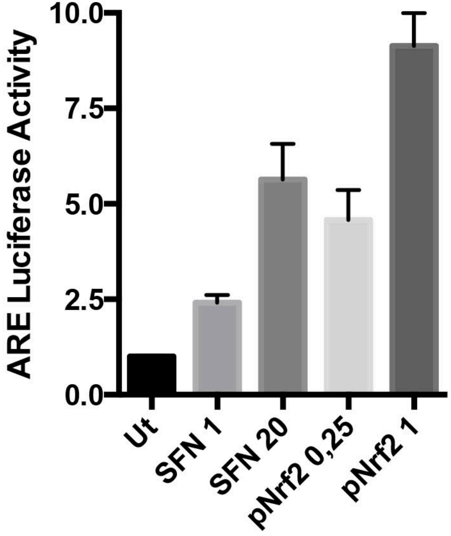

Supplementary Figure 4. Activation of Nrf2 measured by Luciferase assay.
HEK293 cells were transfected with an ARE-luciferase reporter assay. Cells were then treated with SFN in different concentrations (1-20 µM) or co-transfected with an expression plasmid for Nrf2 (pNrf2) in two different concentrations (0.25µg and 1µg). Luciferase activity was then measured to estimate Nrf2 activity. Graph displays means and s.e.m. Data is from one experiment.
See also Fig 1.

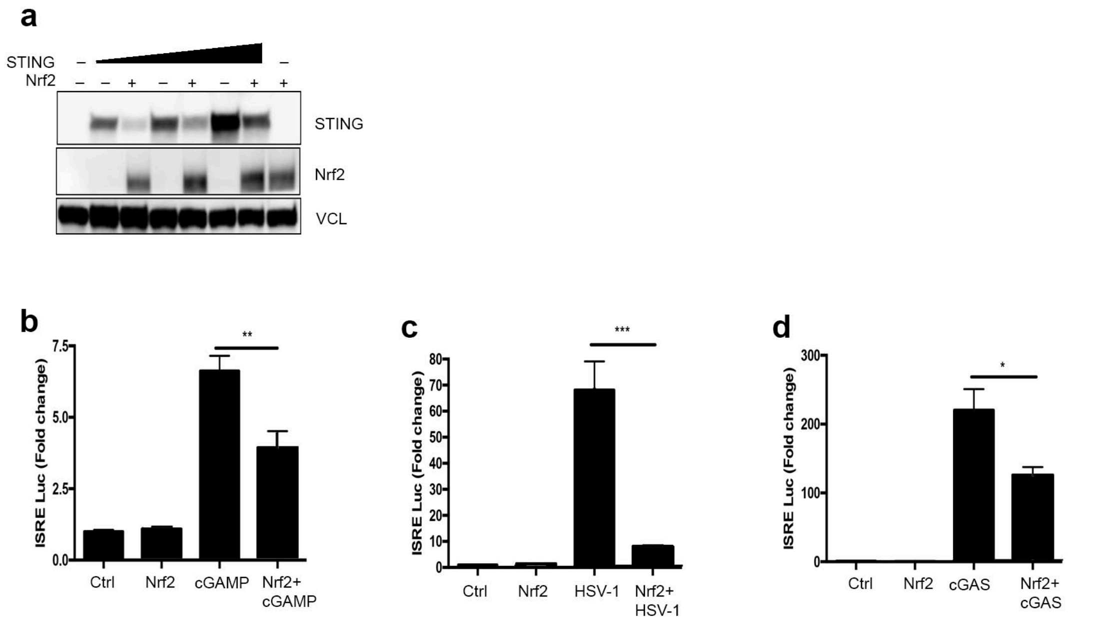

Supplementary Figure 5. Nrf2 affects STING expression and induction of IFN in HEK293 cells. (**a**) HEK293 cells were transfected with different amounts of STING expressing plasmids either alone or in combination with an expression plasmid for Nrf2. Lysates were then immunoblotted for STING, Nrf2, and Vinculin (VCL) as control. Blot is representative of two independen experiments. (**b-d**) HEK293 cells stably expressing STING were transfected with an Nrf2 expression plasmid or with a control plasmid as well as with an ISRE reporter plasmid. Cells were then treated with cGAMP(b), HSV-1 (c), or transfected with a cGAS expressing plasmid. Lysates were analyzed for luciferase activity. Data are represenative of two independent experiments. Students *t*-test was used to determine statistical significance.

See also Fig. 1 and Fig. 3.

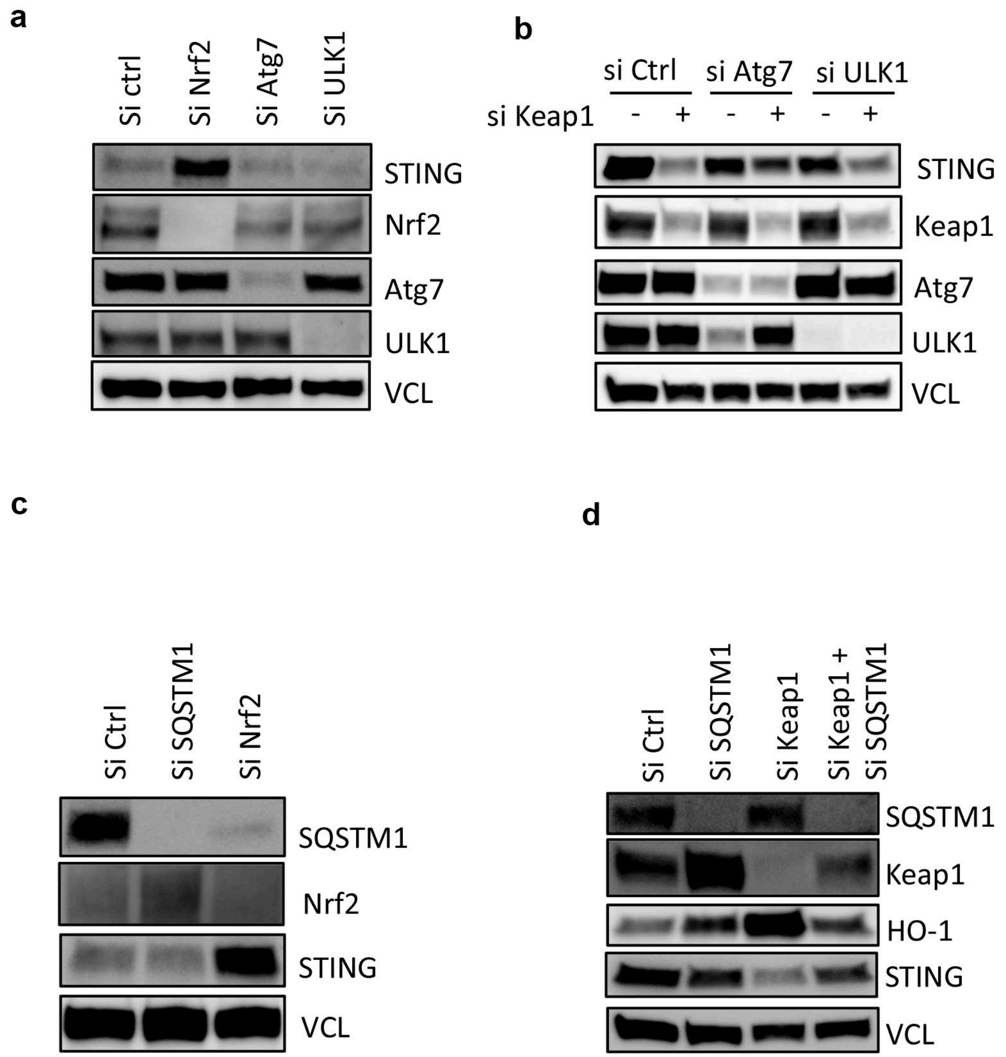

Supplementary Figure 6. Repression of STING by Nrf2 is independent on autophagy.
a) The indicated proteins were silenced with siRNA in A549 cells. Cell lysates were then analyzed by western blotting using vinculin (VCL) as loading control.
b) HaCat cells were treated with si RNA specific for Keap1 in combination with siRNA specific for either Atg7 or ULK1 as indicated. Cells were then analysed by western blotting.
c) A549 cells were treated with siRNA as indicated. Cells were then analyzed by western blotting.
d) HaCat cells were treated with siRNA for SQSTM1, Keap1, or both. Cells were then analyzed by western blotting. All blots are representative of two or more independent experiments.
See also Fig 1.

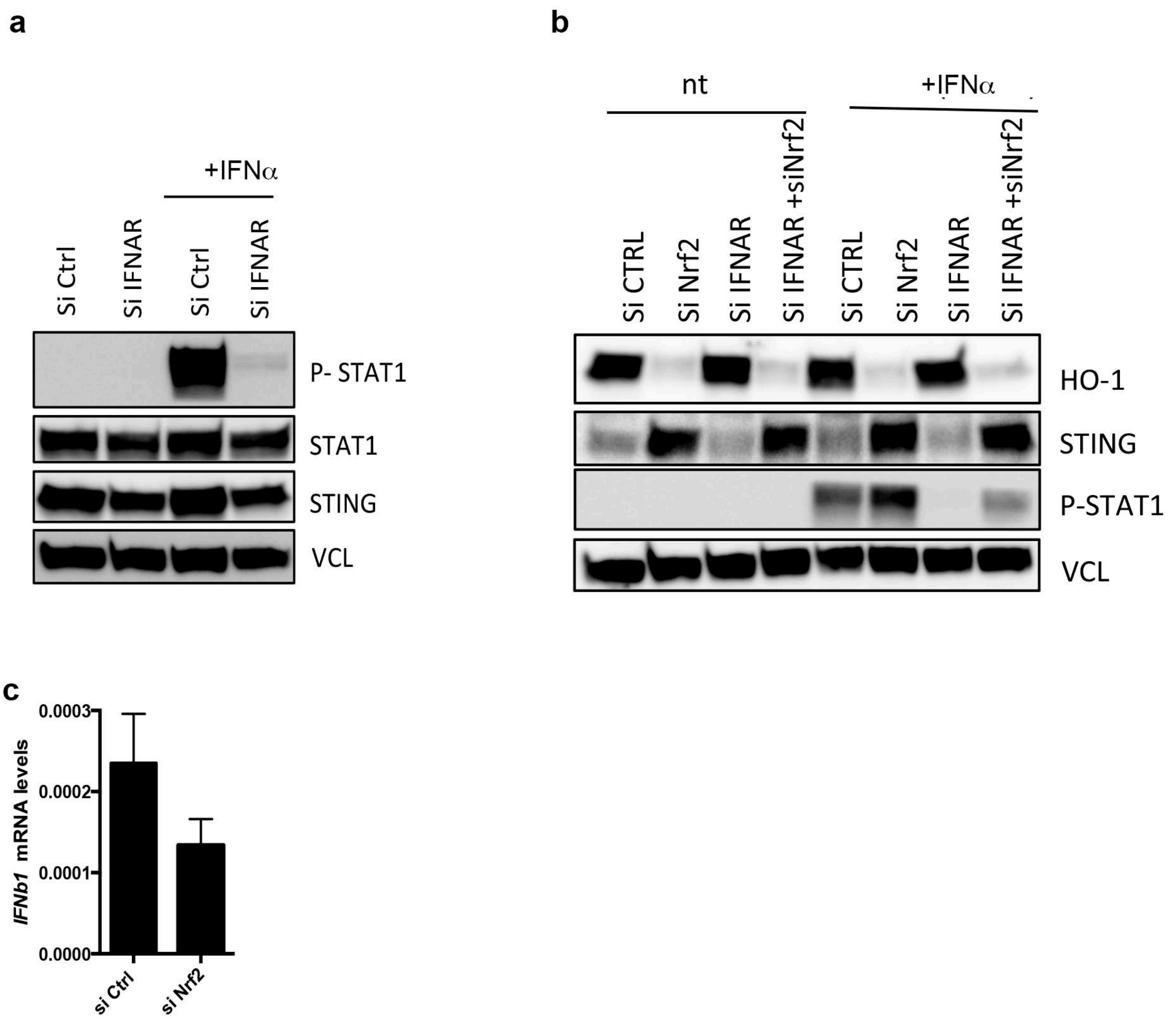

Supplementary Figure 7. Repression of STING by Nrf2 is independent of type I IFN signaling.
(**a**) HaCat cells were treated with siRNA specific for IFNAR before treatment with IFNα for 30 min. Cells were then analysed for expression of STING and for pSTAT1 by western blotting.
(**b**) A549 cells were treated with siRNA specific for Nrf2, IFNAR, or both, before treatment with IFNα for 30 min. Cells were then analyzed by western blotting for indicated proteins.
(**c**) A549 cells were treated with indicated siRNAs for 48 hours and then analyzed for expression of IFNβ by qPCR. Graph displays mean and s.e.m.
Blots and graph are representative of two independent experiments.
See also Fig 1.

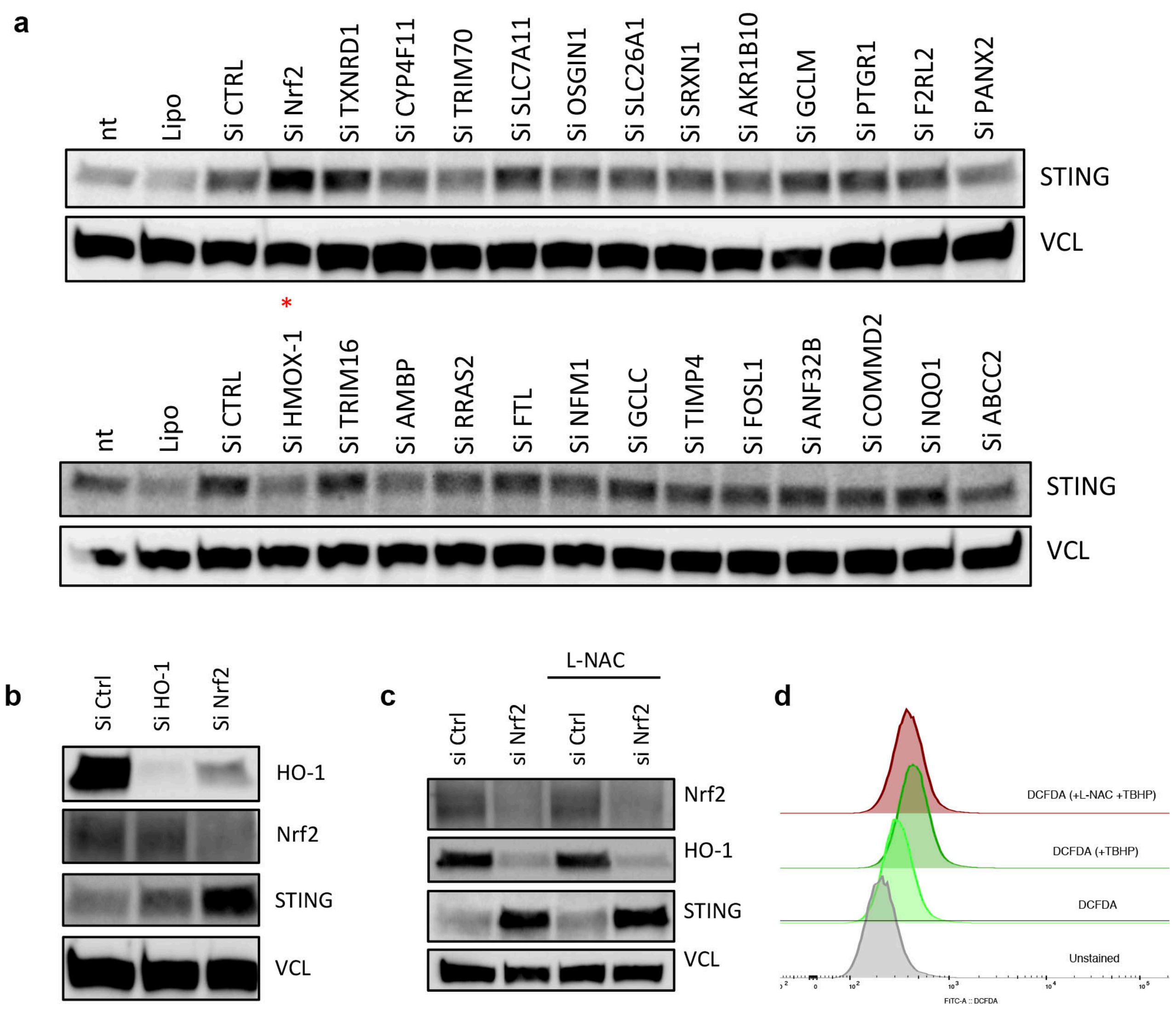

Supplementary Figure 8. Repression of STING expression by Nrf2 is independent of ROS accumulation.
a) A549 cells were transfected with a Cherry pick Nrf2 siRNA library from Dharmacon for 48h. Whole-cell extracts were then analysed for STING protein expression by immunoblotting.
b) A549 cells were transfected with siRNA against HO-1 or Nrf2 for 48h.
Whole-cell extracts were analysed for STING protein expression by immunoblotting.
c) A549 cells were transfected with siRNA against Nrf2 for 48h in the presence or not of the reactive oxygen species (ROS) scavenger L-NAC (10mM). Whole-cell extracts were analysed for STING, HO-1 and Nrf2 by immunoblotting. (**d**) The ROS scavenging effect of L-NAC was assessed in A549 cells stimulated with the ROS inducer TBHP in presence or not of L-NAC (10mM). Intracellular ROS accumulation was measured using the fluorescent DCFDA probe and flow cytometry analysis.Blots display results from one experiment.
See also Fig 1.

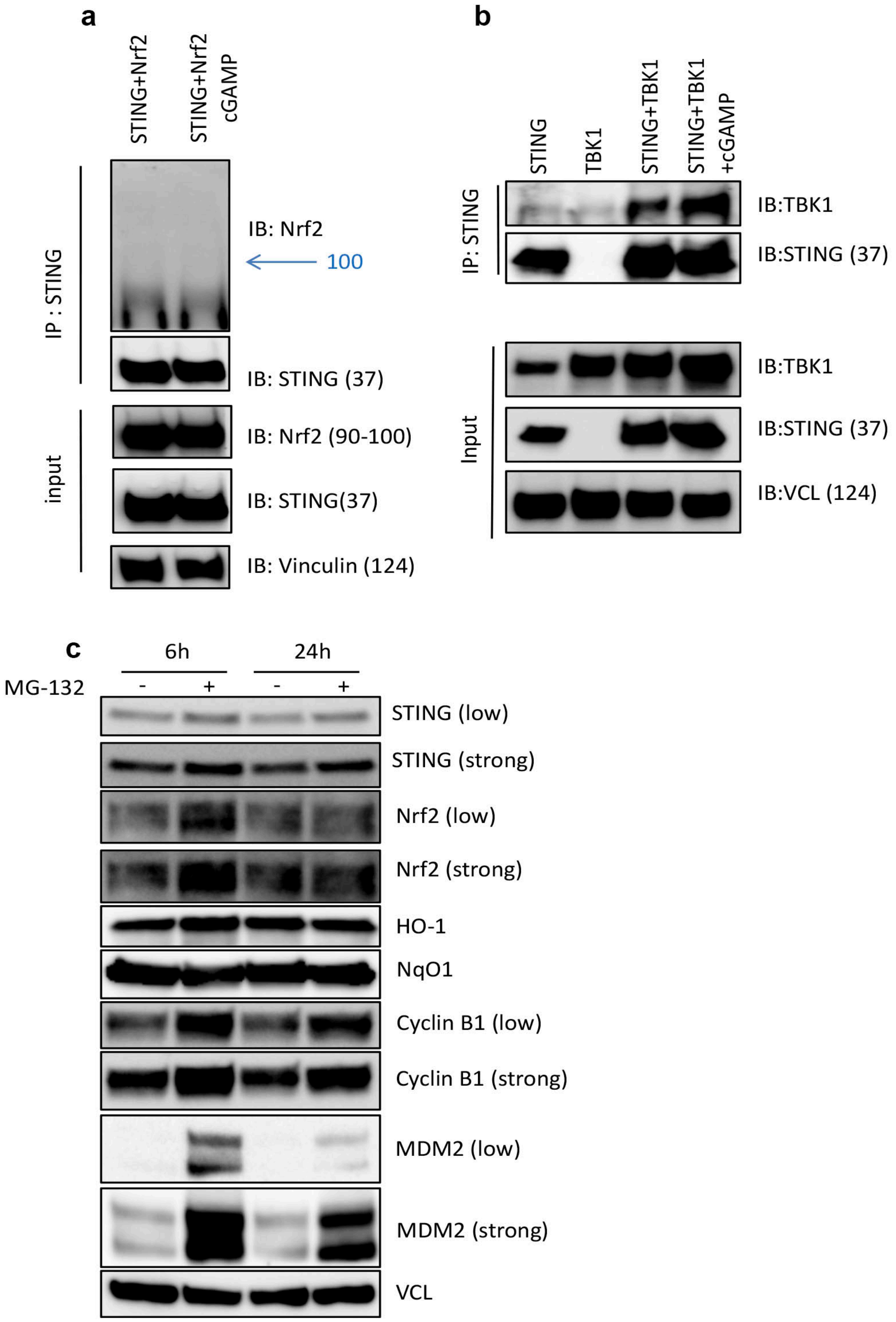

Supplementary Figure 9. Co-precipitation of STING and Nrf2, and the role of proteosomal degradation of STING

a) Immunoprecipitation using a STING-specific antibody was performed on cell lysates from HEK293 cells overexpressing both STING and Nrf2. The Input and the precipitate was then blotted for STING and Nrf2. Lysates from both untreated and cGAMP treated (4µg.mL^{-1}, 6 hours) HEK293 cells were used. Blue arrow indicated 100 kDa and the approximate position of Nrf2.

b) Immunoprecipitation using a STING-specific antibody was performed on lysates from HEK293 cells overexpressing both STING and TBK1. The input and precipitate was then blotted for STING and TBK1 as indicated. Vinculin (VCL) was used as loading control.

Lysates from both untreated and cGAMP treated (4µg.mL^{-1}, 6 hours) HEK293 cells were used. Blots are representative of two independent IP experiments.

c) A549 cells were treated with the proteosomal inhibitor MG-132 (10µM) for either 6 or 24 hours. Cell lysates were then analyzed by western blotting for the indicated proteins. Blot is representative of two independent experiments.

See also fig 3.

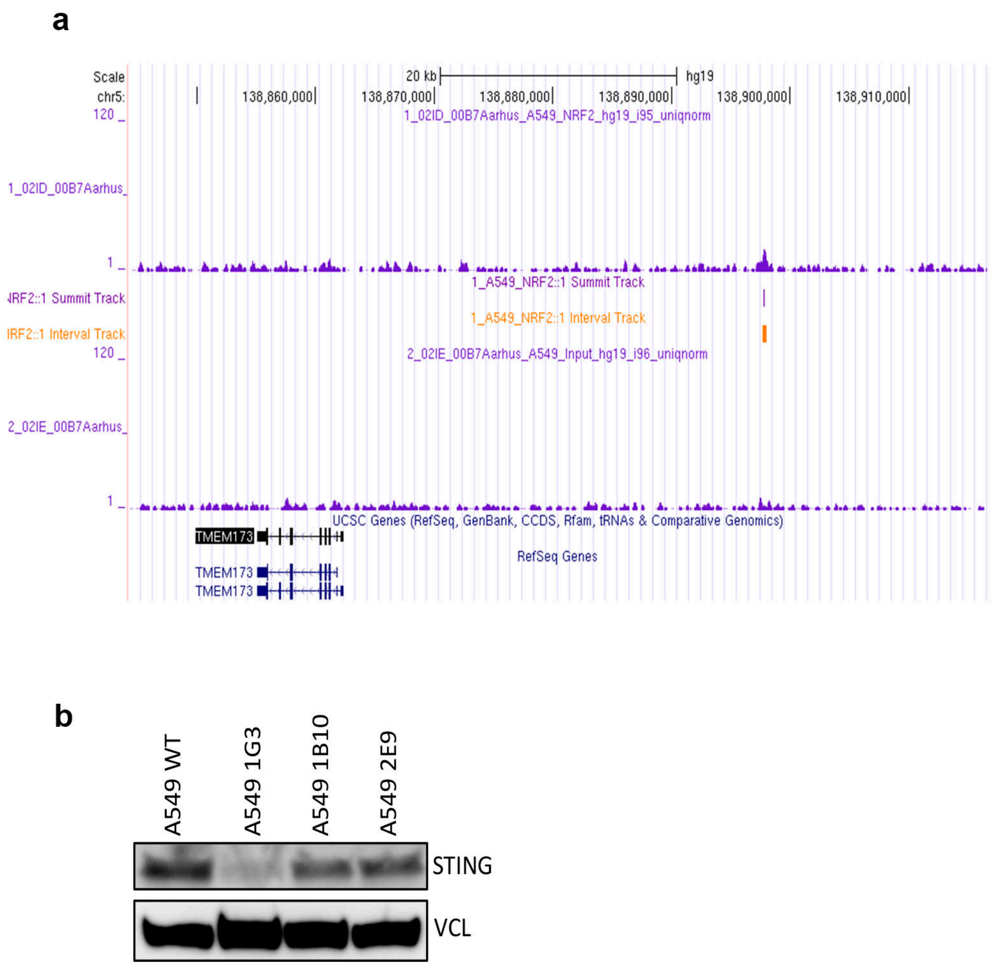

Supplementary Figure 10. Nrf2 peak detected in Nrf2 ChIP-seq with p-value cutoff 1e-3 does not mediate repression of STING expression.

(**a**) UCSC browser screen shot of the RNA read pile-up from the Nrf2 ChIP-seq in proximity to *TMEM173.*

(**b**) Western blotting displaying the expression of STING in WT A549 cells compared with three A549 cell clones where the genomic region of the Nrf2 peak was eliminated by CrisprCas9 gene editing. Vinculin (VCL) was used as loading control.

Data are from one experiment.

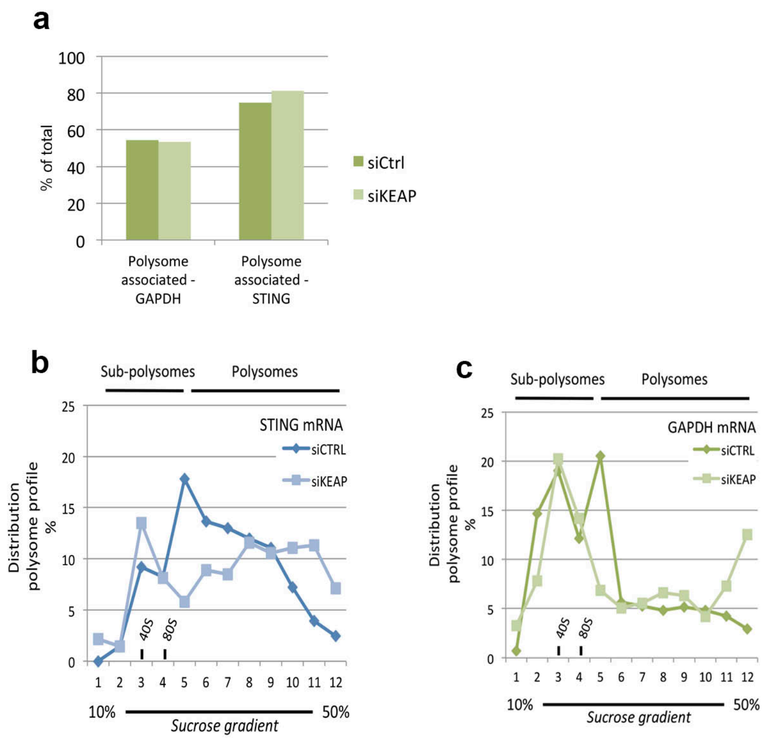

Supplementary Figure 11. Polysome profiling of STING and GAPDH mRNAs. HaCat cells were either silenced for KEAP1 using siRNA (siKEAP) or treated with control siRNA (siCTRL).
After 48 hours, cell lysates were prepared for 10-50% sucrose gradient fractionation in the presence of 0.1 mg/ml cycloheximide. Total RNA from 12 fractions was isolated and subjected to RT-qPCR.
a) Quantification of STING and GAPDH mRNA associated with polysomes (fraction 5-12), % of total.
b-c) Distribution of STING and GAPDH mRNA thoughout the sucrose gradient, % of total amount. Data are from one experiment perfromed in dublicate.
See also Fig. 2.

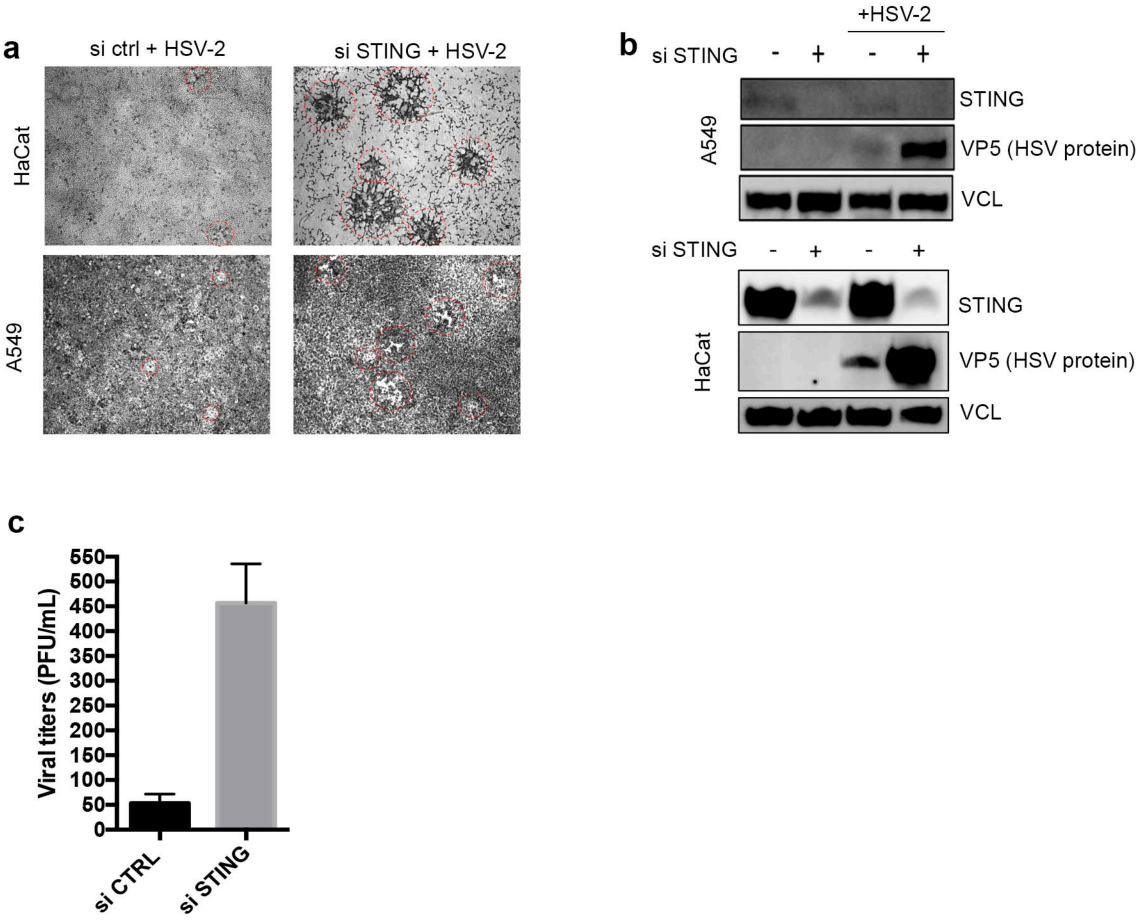

Supplementary Figure 12. STING is important for viral replication in A549 and HaCat cells.
(**a** and **b**) HaCat and A549 cells were treated with control siRNA (ctrl) or STING siRNA for 48 hours. Cells were then infected with HSV-2 (MOI 0.01 for HaCat and 0.01 for A549). Cells were then incubated overnight.
(**a**) Bright-field photo of cell cultures infected with HSV-2 and treated with either ctlr or STING siRNA.
(**b**) Immunoblot of cell lysates blotted for STING, the HSV-2 protein VP5, and for Vinculin (VCL) as loading control.
(**c**) Cell-medium containing progeny virus was collected from experiment with A549 cells in a). Viral titers were determined using Vero-cell based plaque assay. Graph displays mean and s.e.m. Data are representative of two independen experiments.
See also Fig. 3.

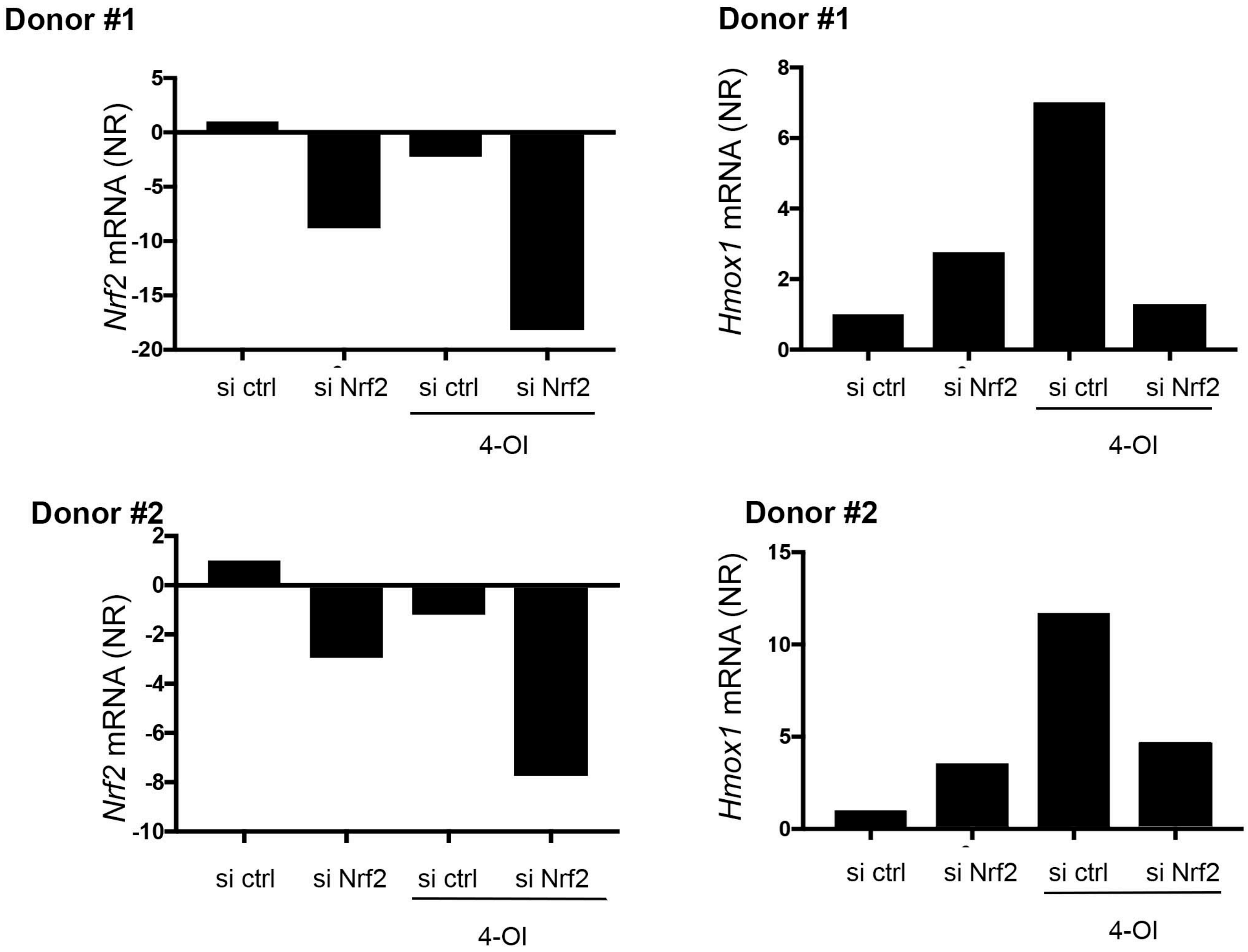

Supplementary Figure 13.
Human primary monocytes were treated with siRNA twice during differentiation into human monocyte derived macrophages using M-CSF. Cells were then either left untreated (Nt) or treated with 4-OI for 48 hours. Cellular RNA was then extracted and analyzed by qPCR. Levels were normalized to Nt (ctrl si RNA). Data are from one experiment using two independen healthy donors. Bars indicate mean.
See also Fig 4.

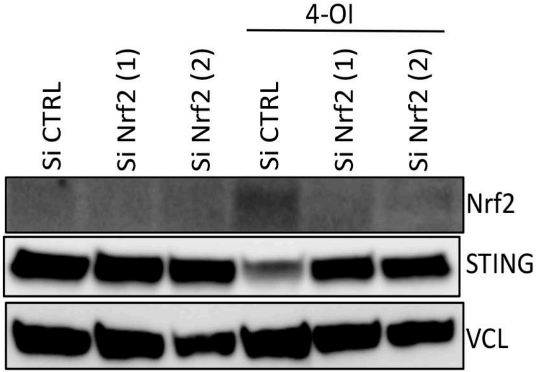

Supplementary Figure 14. Repression of STING by 4-OI depends on Nrf2
HaCat cells were treated with control siRNA (si ctrl) or two different siRNA sequences (1 and 2) both targeting Nrf2 for 48 hours. Cells were then stimulated with 4-OI for an additional 48 hours. Cells were then lysed and analysed by western blotting for Nrf2, STING and Vinculin (VCL) as loading control. Blot is from one experiment using different siRNAs.
See also Fig. 4.

Supplementary Figure 15

Uncropped WBs

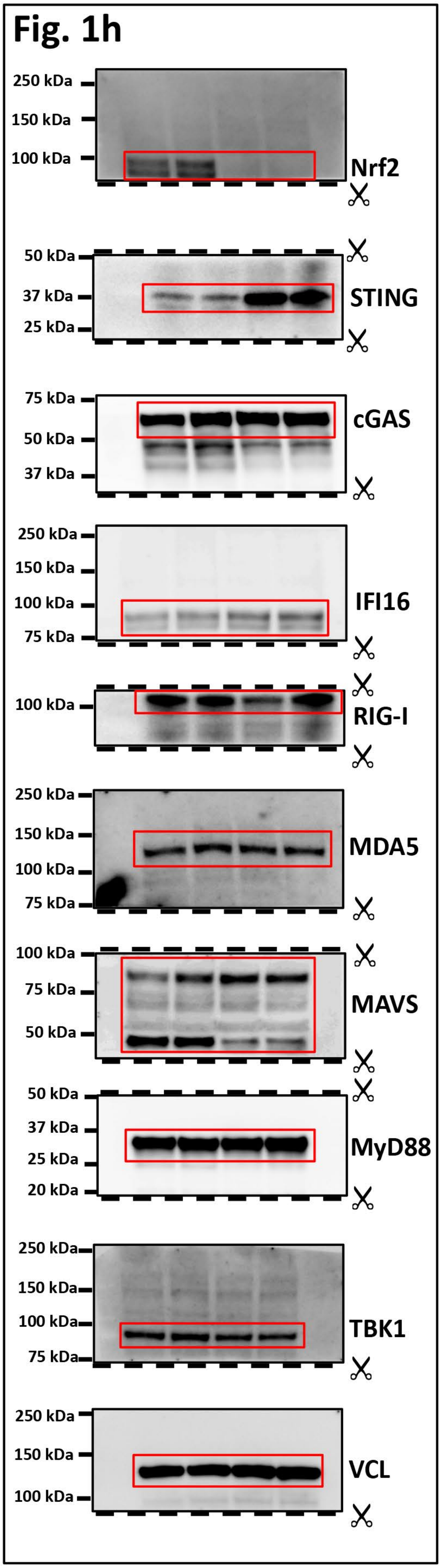

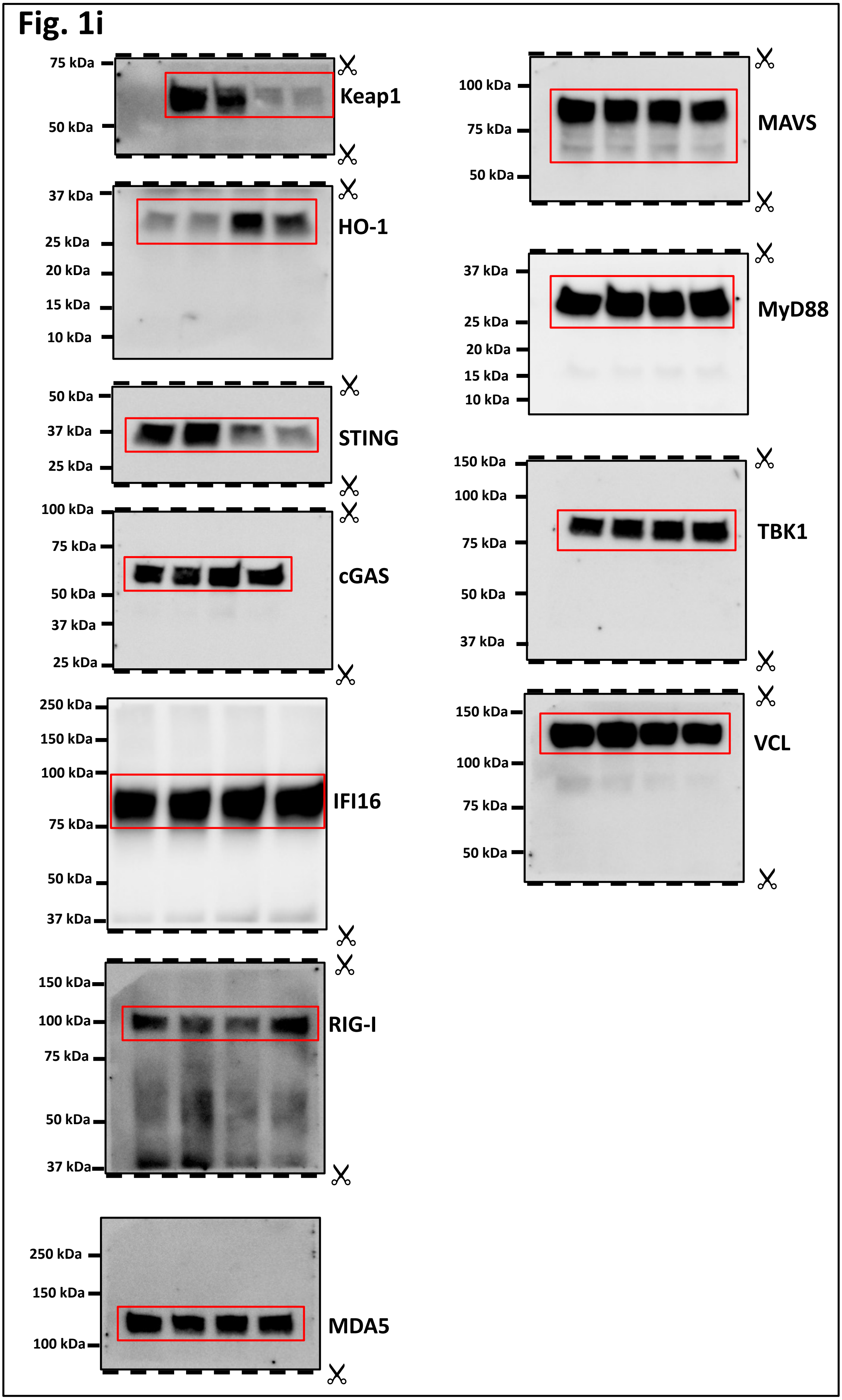
Fig. 1i
Keap1
HO-1
STING
cGAS
IFI16
RIG-I
MDA5
MAVS
MyD88
TBK1
VCL
75 kDa
50 kDa
37 kDa
25 kDa
20 kDa
15 kDa
10 kDa
100 kDa
250 kDa
150 kDa

Fig. 1n

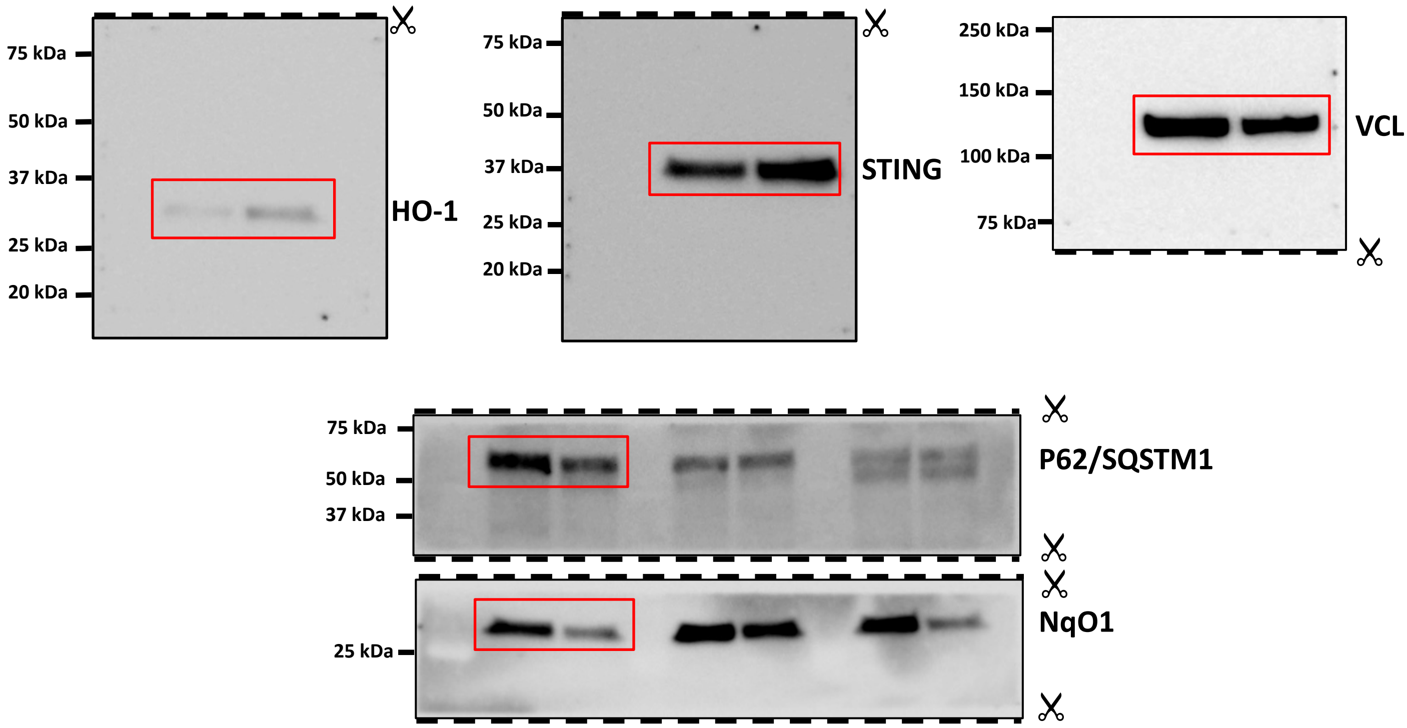

Fig. 1p

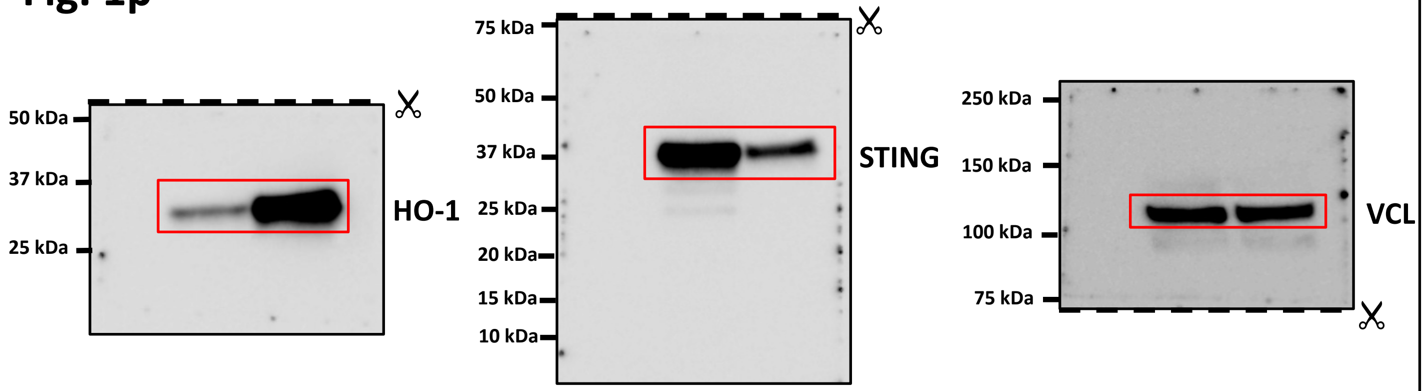

Fig. 1r

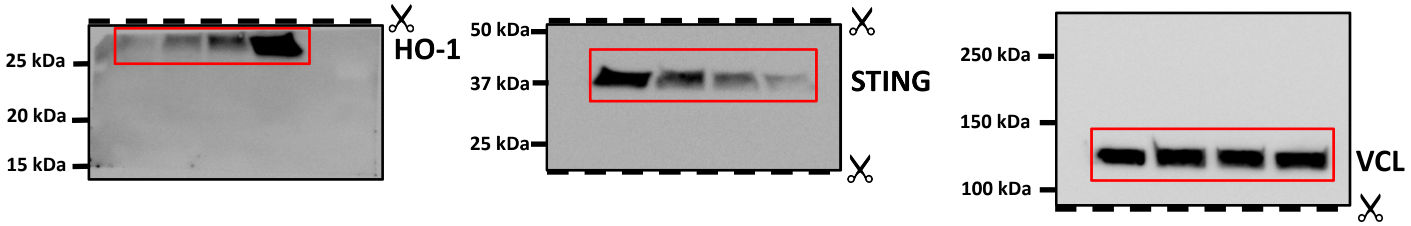

Fig. 3a

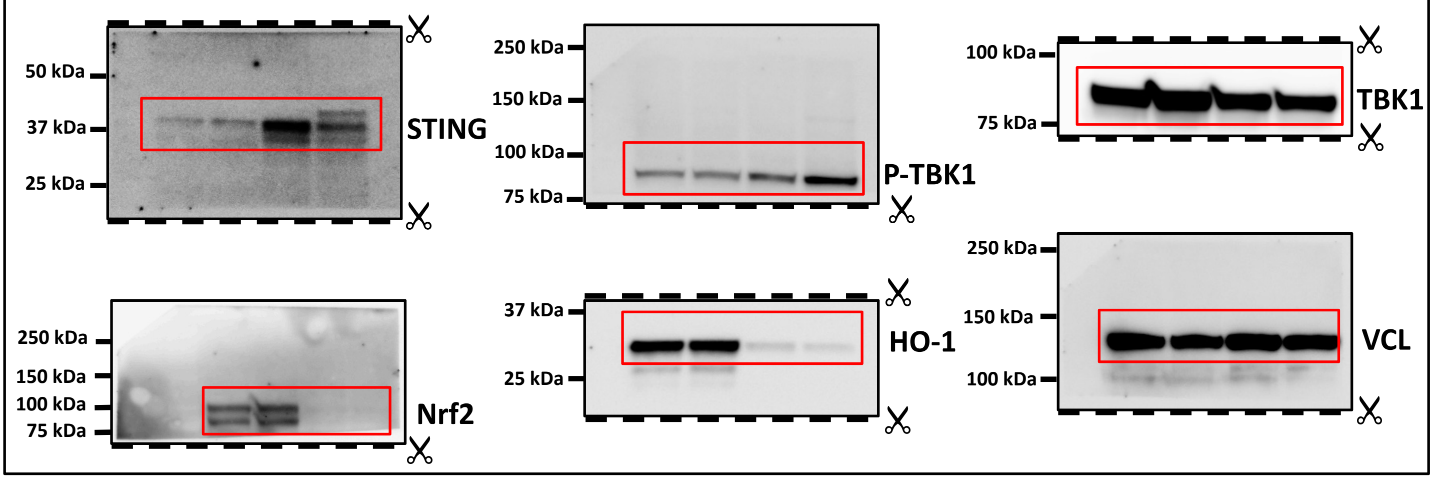

Fig. 3b

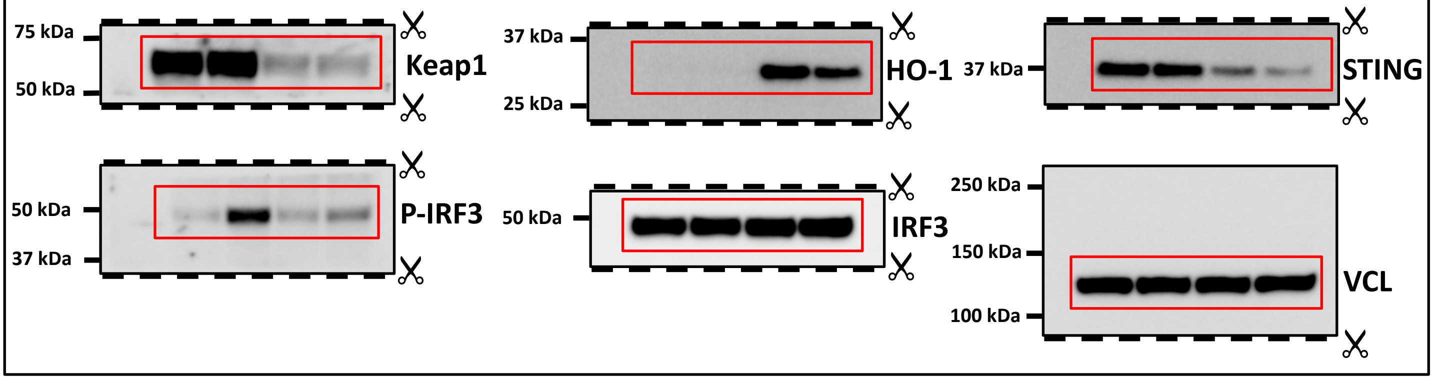

Fig. 3d

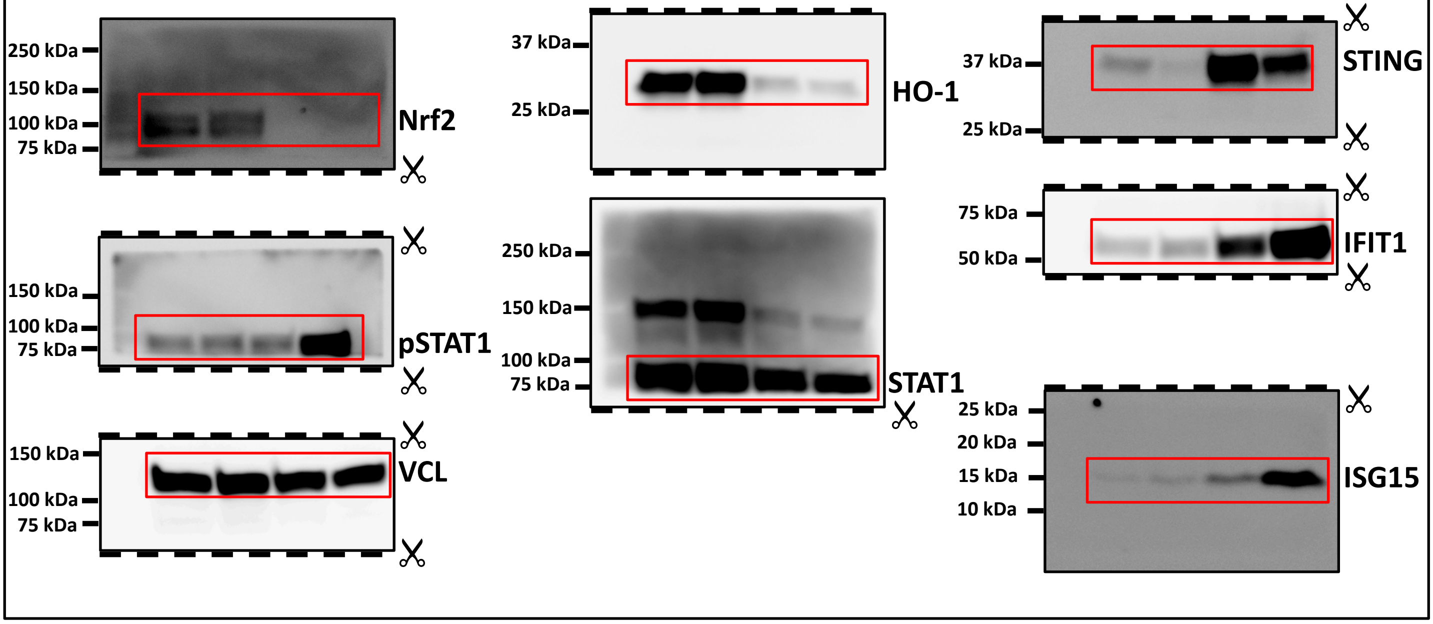

Fig. 3e

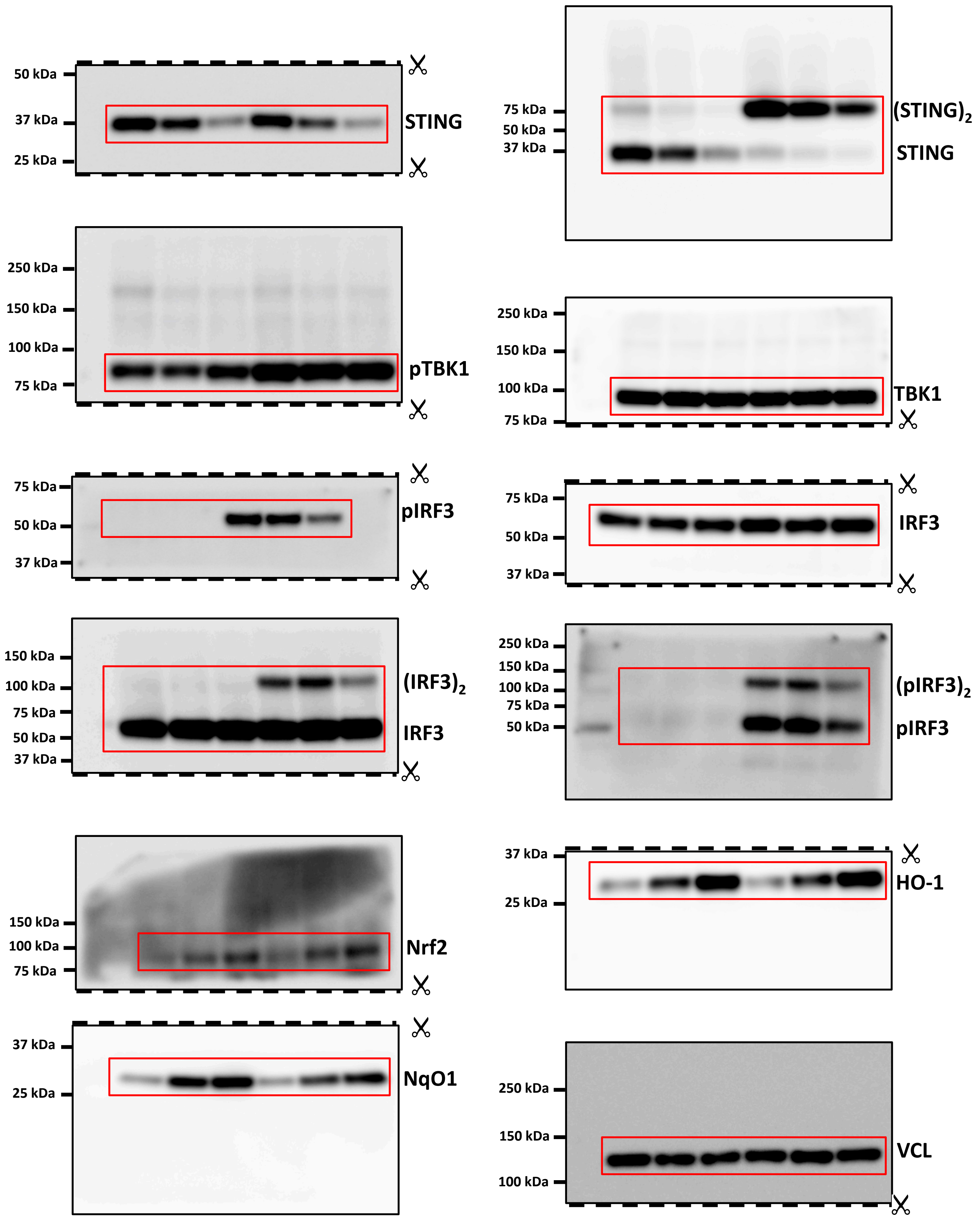

50 kDa
37 kDa
25 kDa
STING
75 kDa
50 kDa
37 kDa
(STING)2
STING
250 kDa
150 kDa
100 kDa
75 kDa
pTBK1
250 kDa
150 kDa
100 kDa
75 kDa
TBK1
75 kDa
50 kDa
37 kDa
pIRF3
75 kDa
50 kDa
37 kDa
IRF3
150 kDa
100 kDa
75 kDa
50 kDa
37 kDa
(IRF3)2
IRF3
250 kDa
150 kDa
100 kDa
75 kDa
50 kDa
(pIRF3)2
pIRF3
150 kDa
100 kDa
75 kDa
Nrf2
37 kDa
25 kDa
HO-1
37 kDa
25 kDa
NqO1
250 kDa
150 kDa
100 kDa
VCL

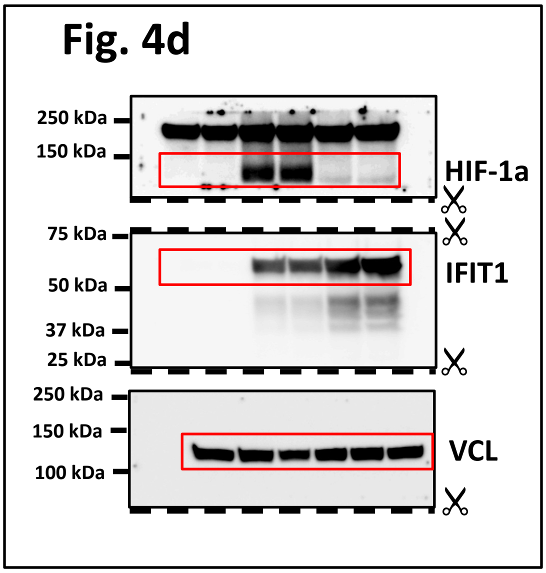
Fig. 4d
250 kDa
150 kDa
HIF-1a
75 kDa
IFIT1
50 kDa
37 kDa
25 kDa
250 kDa
150 kDa
VCL
100 kDa

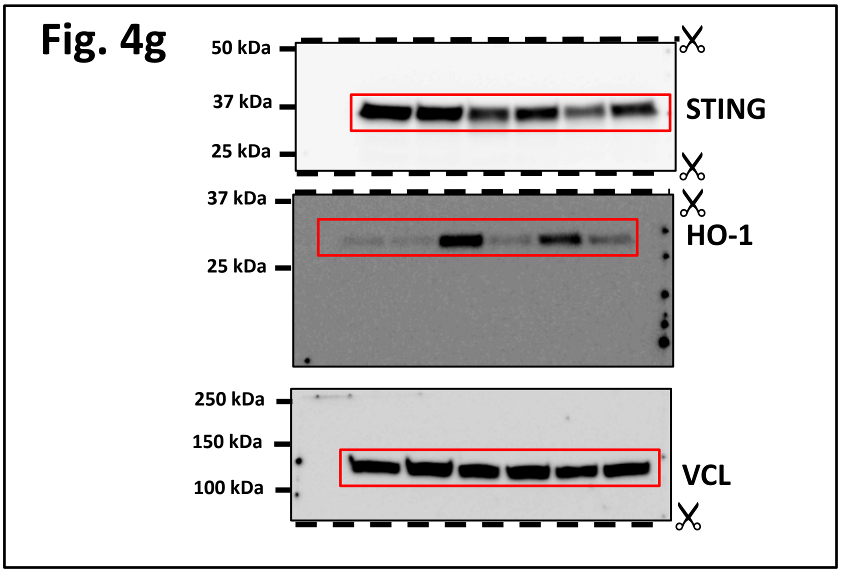
Fig. 4g
50 kDa
37 kDa
STING
25 kDa
37 kDa
HO-1
25 kDa
250 kDa
150 kDa
VCL
100 kDa

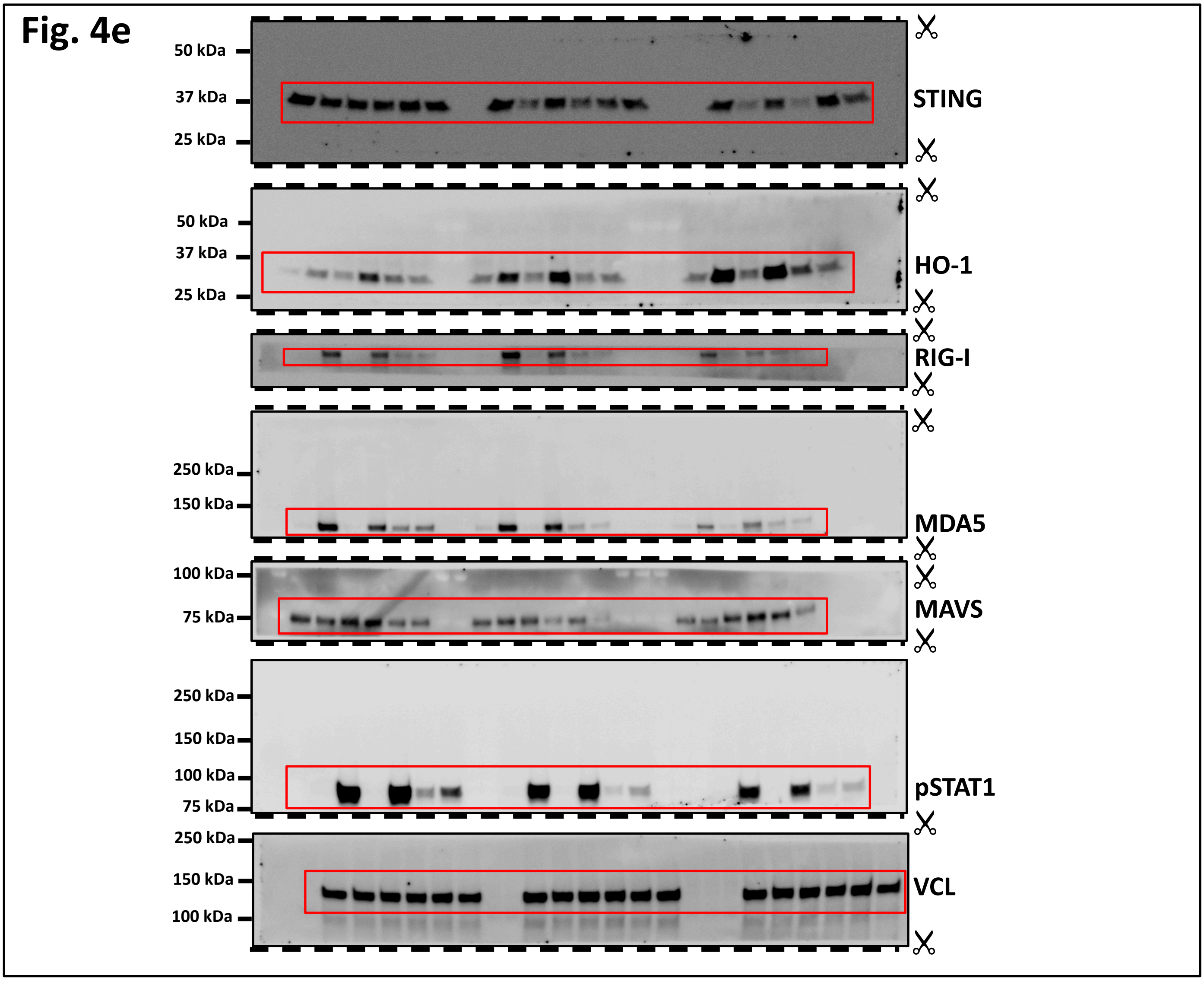
Fig. 4e
50 kDa
37 kDa
STING
25 kDa
50 kDa
37 kDa
HO-1
25 kDa
RIG-I
250 kDa
150 kDa
MDA5
100 kDa
MAVS
75 kDa
250 kDa
150 kDa
100 kDa
pSTAT1
75 kDa
250 kDa
150 kDa
VCL
100 kDa

Fig. 4i

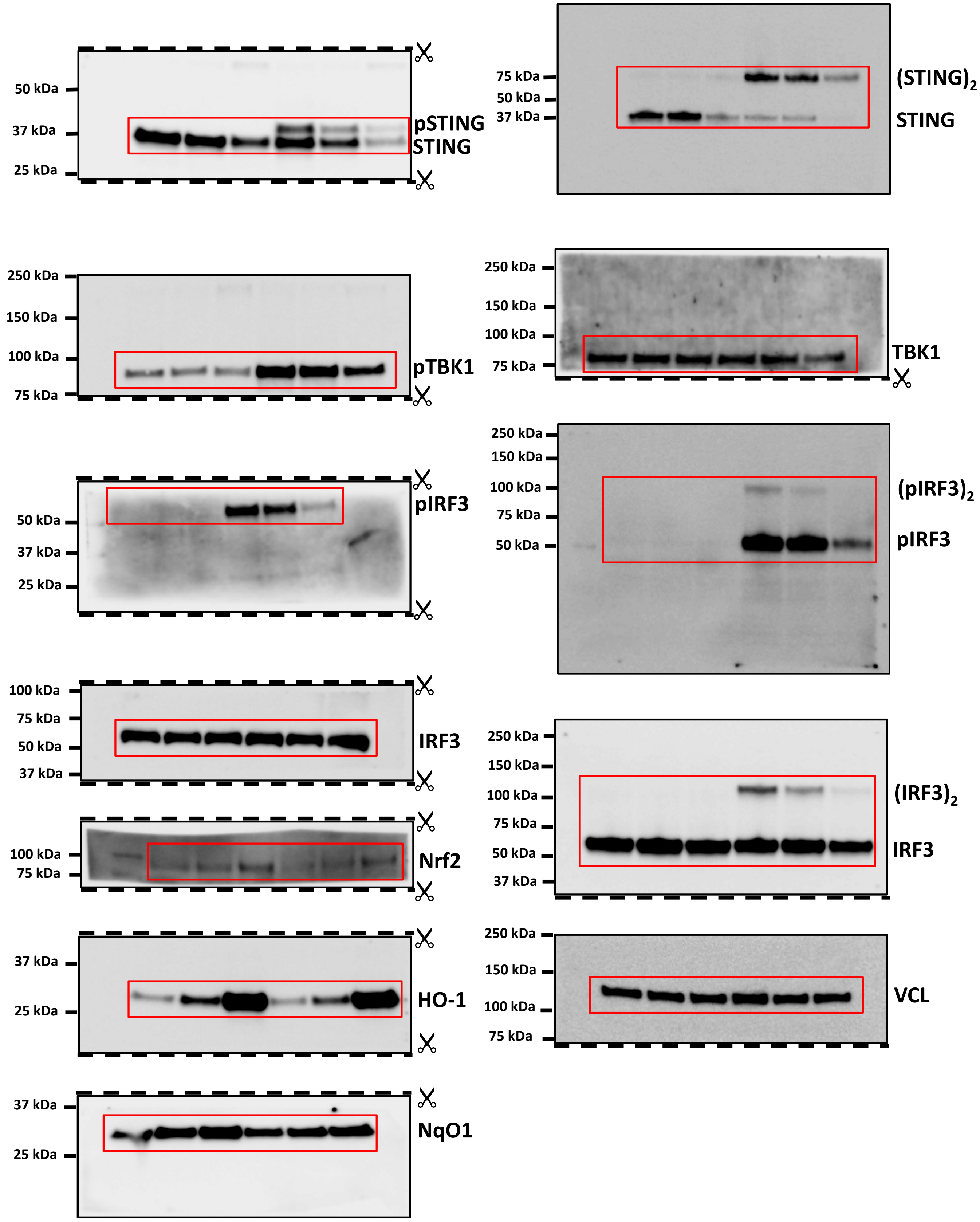
pSTING
STING
50 kDa
37 kDa
25 kDa
(STING)$_2$
STING
75 kDa
50 kDa
37 kDa
pTBK1
250 kDa
150 kDa
100 kDa
75 kDa
TBK1
250 kDa
150 kDa
100 kDa
75 kDa
pIRF3
50 kDa
37 kDa
25 kDa
(pIRF3)$_2$
pIRF3
250 kDa
150 kDa
100 kDa
75 kDa
50 kDa
IRF3
100 kDa
75 kDa
50 kDa
37 kDa
(IRF3)$_2$
IRF3
250 kDa
150 kDa
100 kDa
75 kDa
50 kDa
37 kDa
Nrf2
100 kDa
75 kDa
HO-1
37 kDa
25 kDa
VCL
250 kDa
150 kDa
100 kDa
75 kDa
NqO1
37 kDa
25 kDa

Fig. 4n

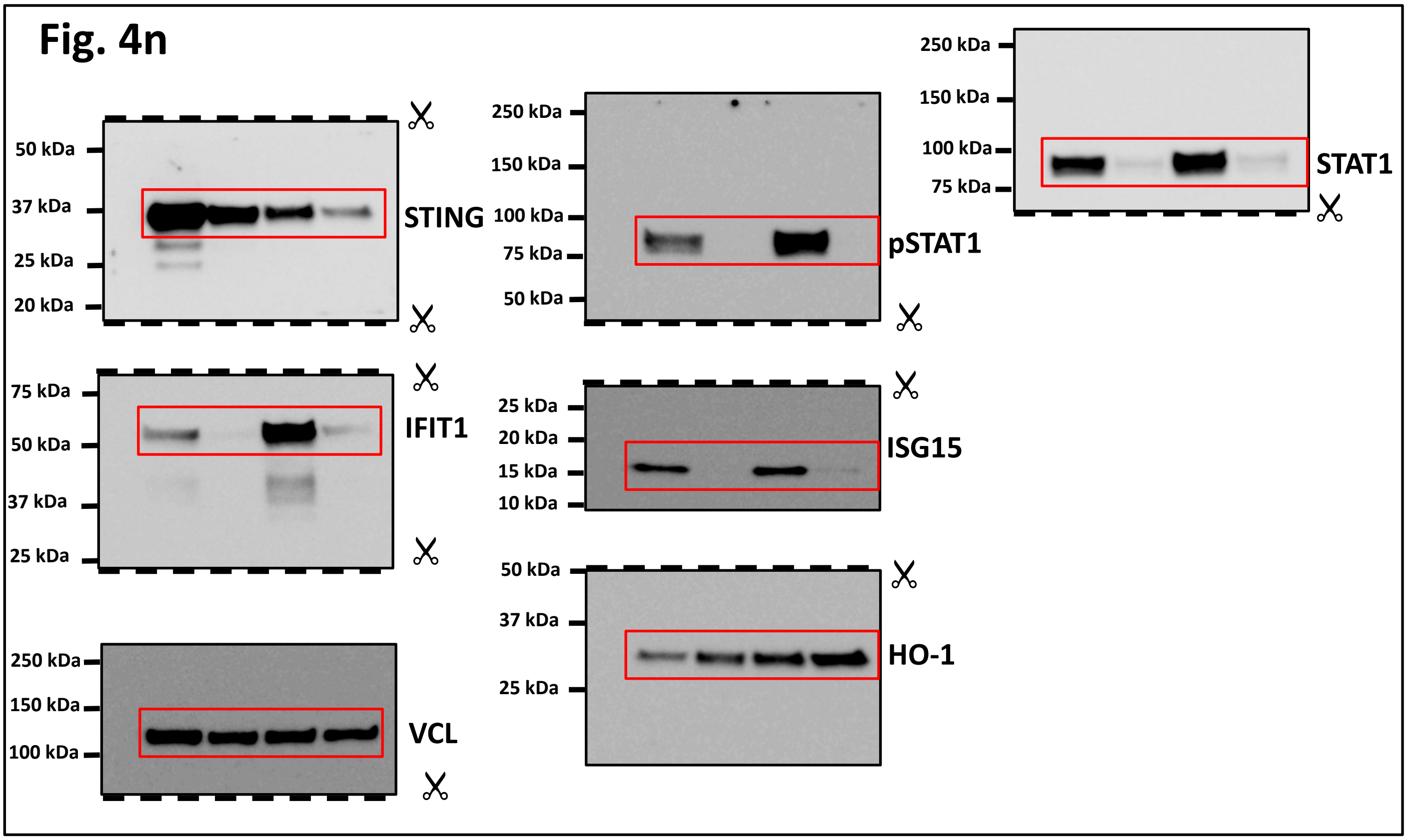
Fig. 4n
50 kDa
37 kDa
25 kDa
20 kDa
STING
250 kDa
150 kDa
100 kDa
75 kDa
50 kDa
pSTAT1
250 kDa
150 kDa
100 kDa
75 kDa
STAT1
75 kDa
50 kDa
37 kDa
25 kDa
IFIT1
25 kDa
20 kDa
15 kDa
10 kDa
ISG15
250 kDa
150 kDa
100 kDa
VCL
50 kDa
37 kDa
25 kDa
HO-1

Fig. 4o

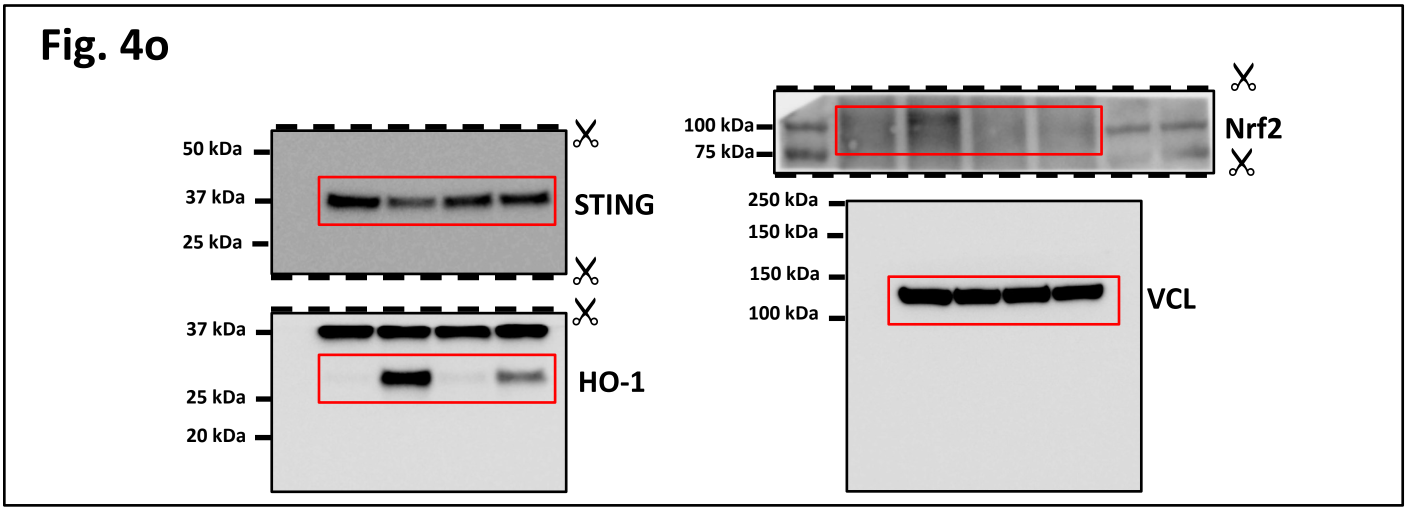
Fig. 4o
50 kDa
37 kDa
25 kDa
STING
37 kDa
25 kDa
20 kDa
HO-1
100 kDa
75 kDa
Nrf2
250 kDa
150 kDa
150 kDa
100 kDa
VCL

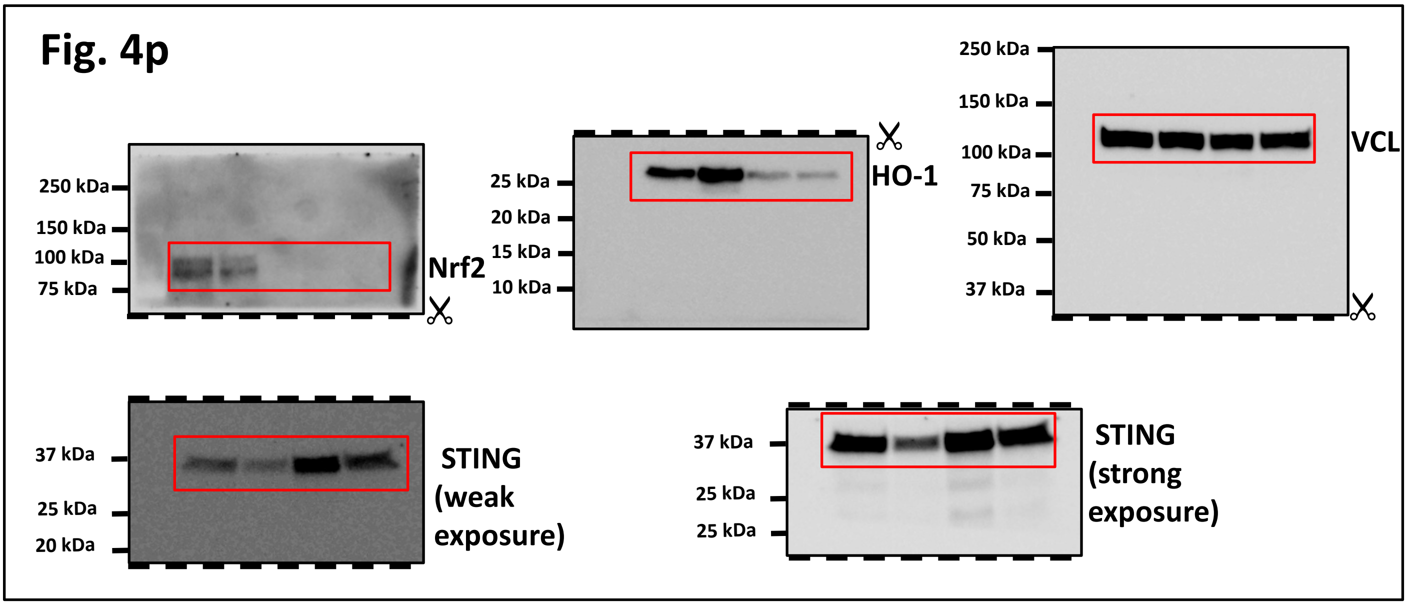
Fig. 4p
250 kDa
150 kDa
100 kDa
75 kDa
Nrf2
25 kDa
20 kDa
15 kDa
10 kDa
HO-1
250 kDa
150 kDa
100 kDa
75 kDa
50 kDa
37 kDa
VCL
37 kDa
25 kDa
20 kDa
STING (weak exposure)
37 kDa
25 kDa
25 kDa
STING (strong exposure)

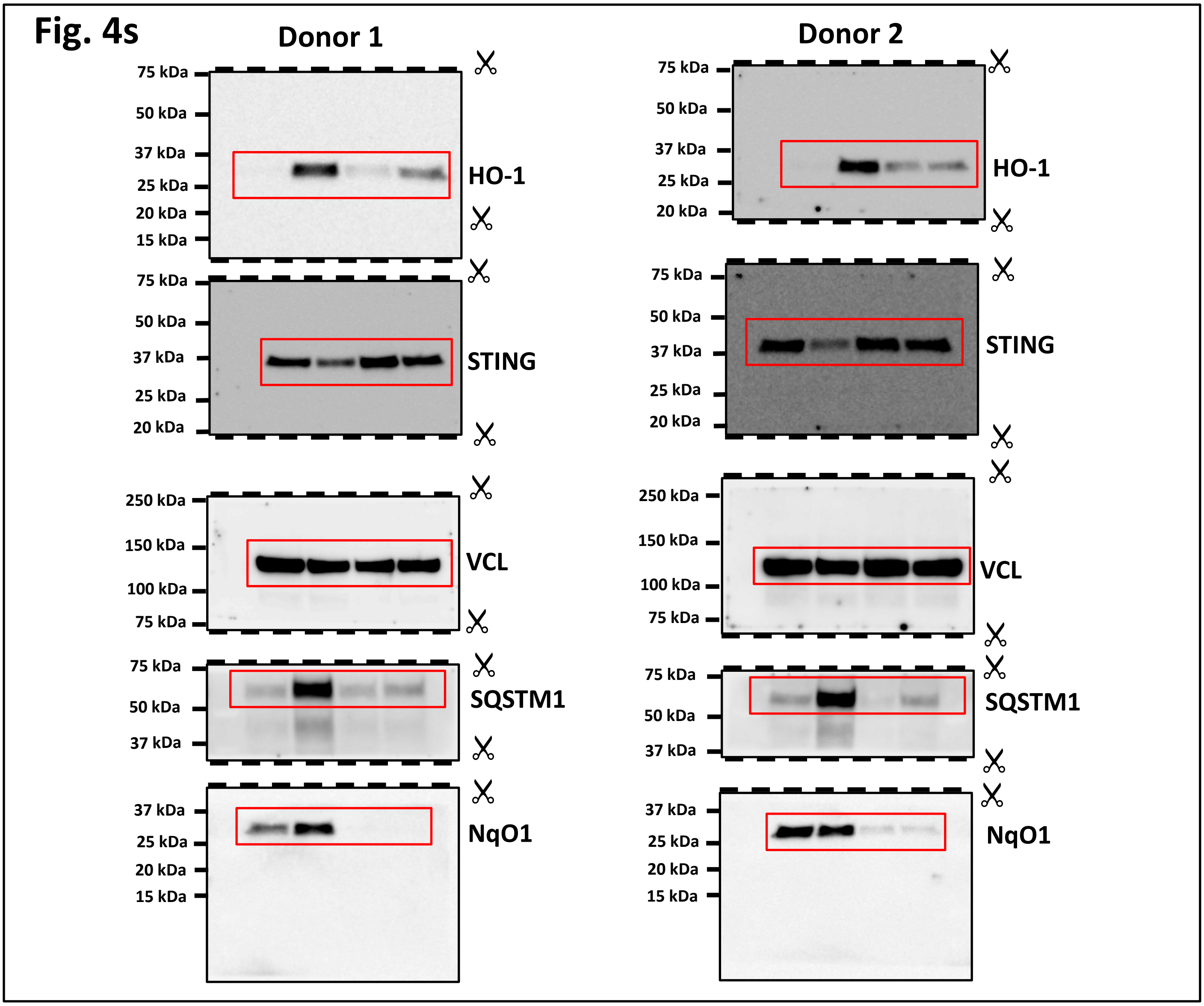
Fig. 4s
Donor 1
Donor 2
75 kDa
50 kDa
37 kDa
25 kDa
20 kDa
15 kDa
HO-1
75 kDa
50 kDa
37 kDa
25 kDa
20 kDa
STING
250 kDa
150 kDa
100 kDa
75 kDa
VCL
75 kDa
50 kDa
37 kDa
SQSTM1
37 kDa
25 kDa
20 kDa
15 kDa
NqO1

Fig. 5e

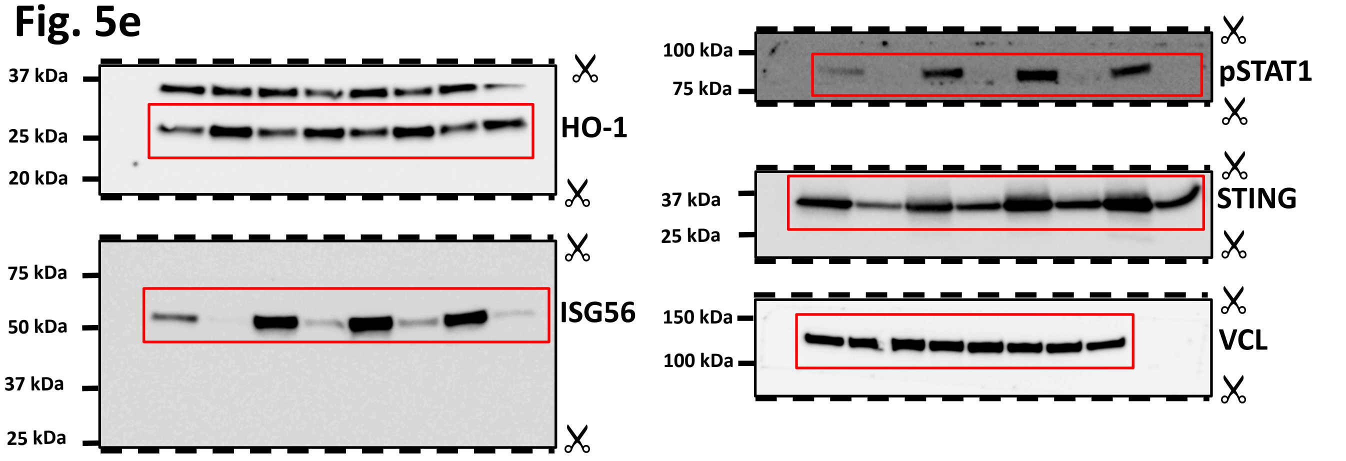

37 kDa
25 kDa
20 kDa
HO-1
75 kDa
50 kDa
37 kDa
25 kDa
ISG56
100 kDa
75 kDa
pSTAT1
37 kDa
25 kDa
STING
150 kDa
100 kDa
VCL

Fig. 5f

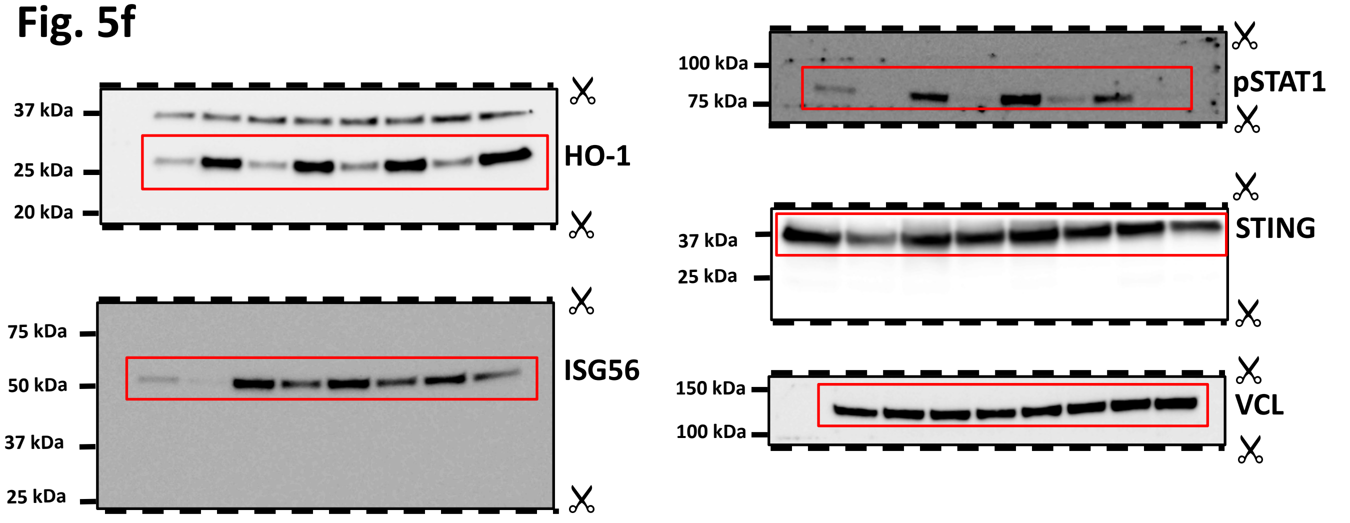

37 kDa
25 kDa
20 kDa
HO-1
75 kDa
50 kDa
37 kDa
25 kDa
ISG56
100 kDa
75 kDa
pSTAT1
37 kDa
25 kDa
STING
150 kDa
100 kDa
VCL

Fig. 5g

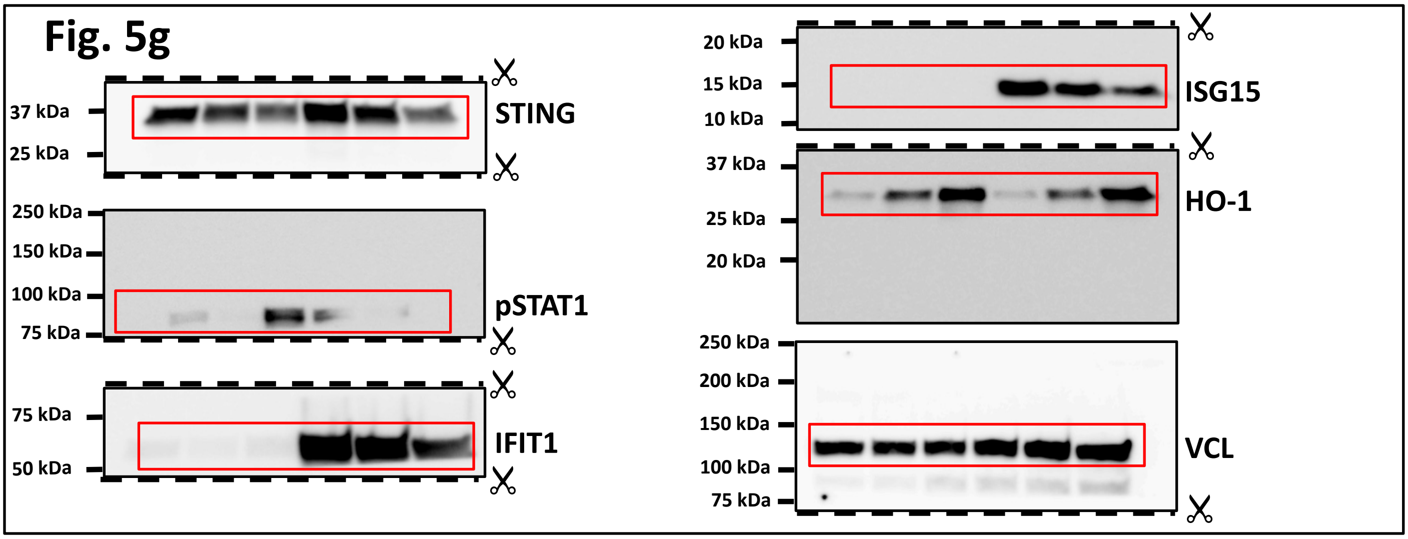

Fig. 5h

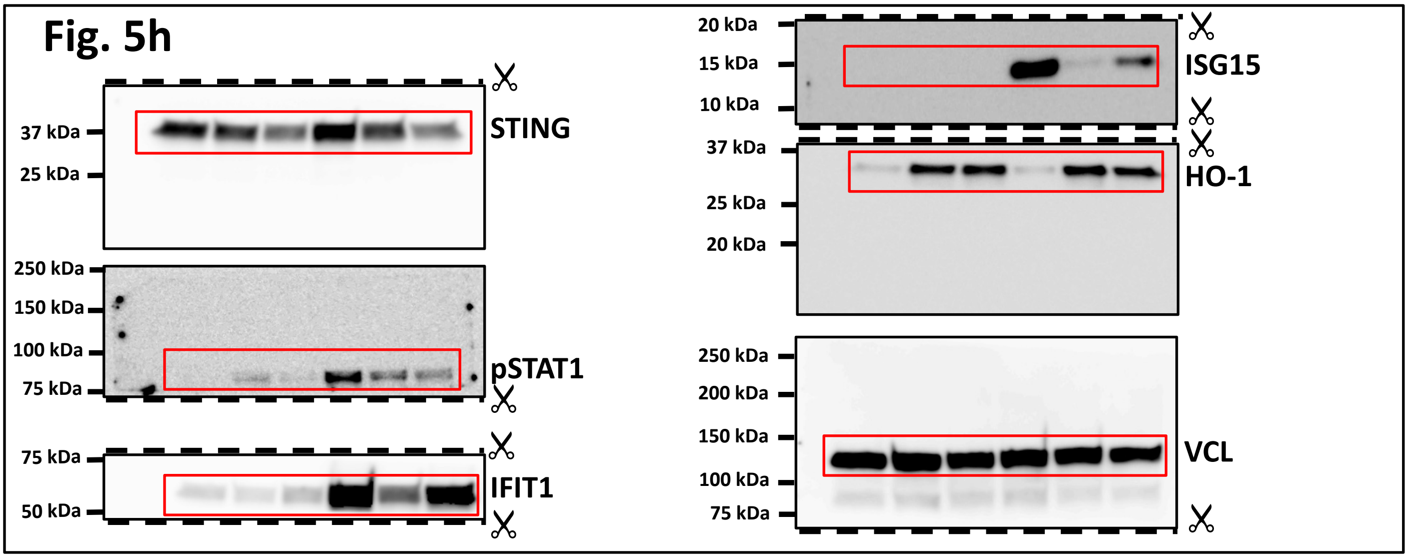

Fig. 5i

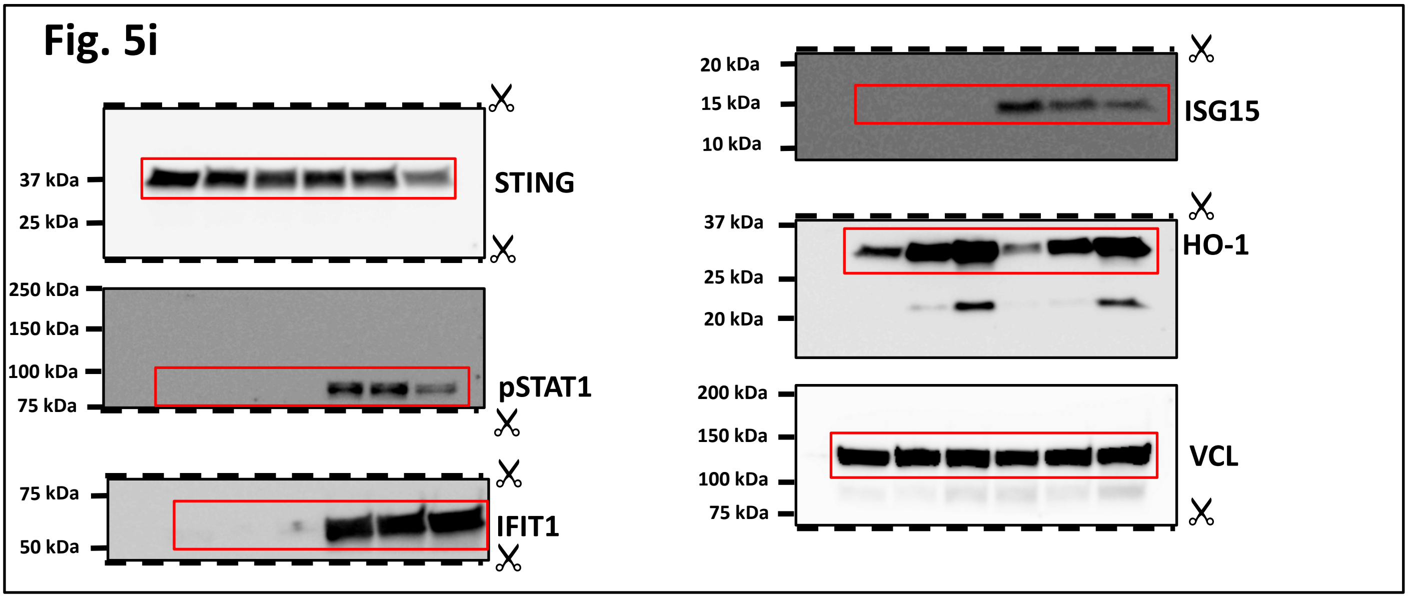

Fig. 5k, l and m

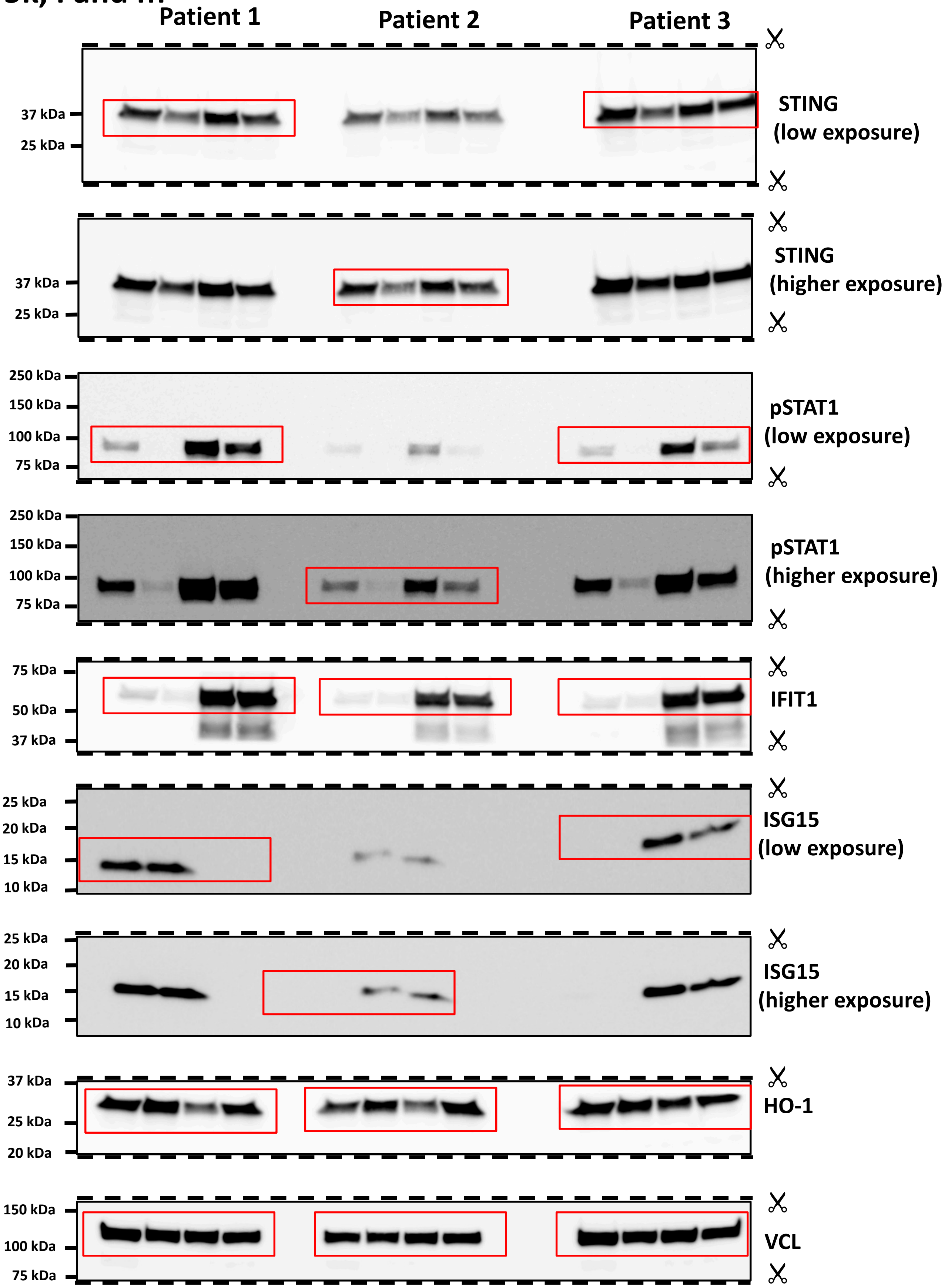

Patient 1
Patient 2
Patient 3
37 kDa
25 kDa
STING
(low exposure)
37 kDa
25 kDa
STING
(higher exposure)
250 kDa
150 kDa
100 kDa
75 kDa
pSTAT1
(low exposure)
250 kDa
150 kDa
100 kDa
75 kDa
pSTAT1
(higher exposure)
75 kDa
50 kDa
37 kDa
IFIT1
25 kDa
20 kDa
15 kDa
10 kDa
ISG15
(low exposure)
25 kDa
20 kDa
15 kDa
10 kDa
ISG15
(higher exposure)
37 kDa
25 kDa
20 kDa
HO-1
150 kDa
100 kDa
75 kDa
VCL

Fig. S2A

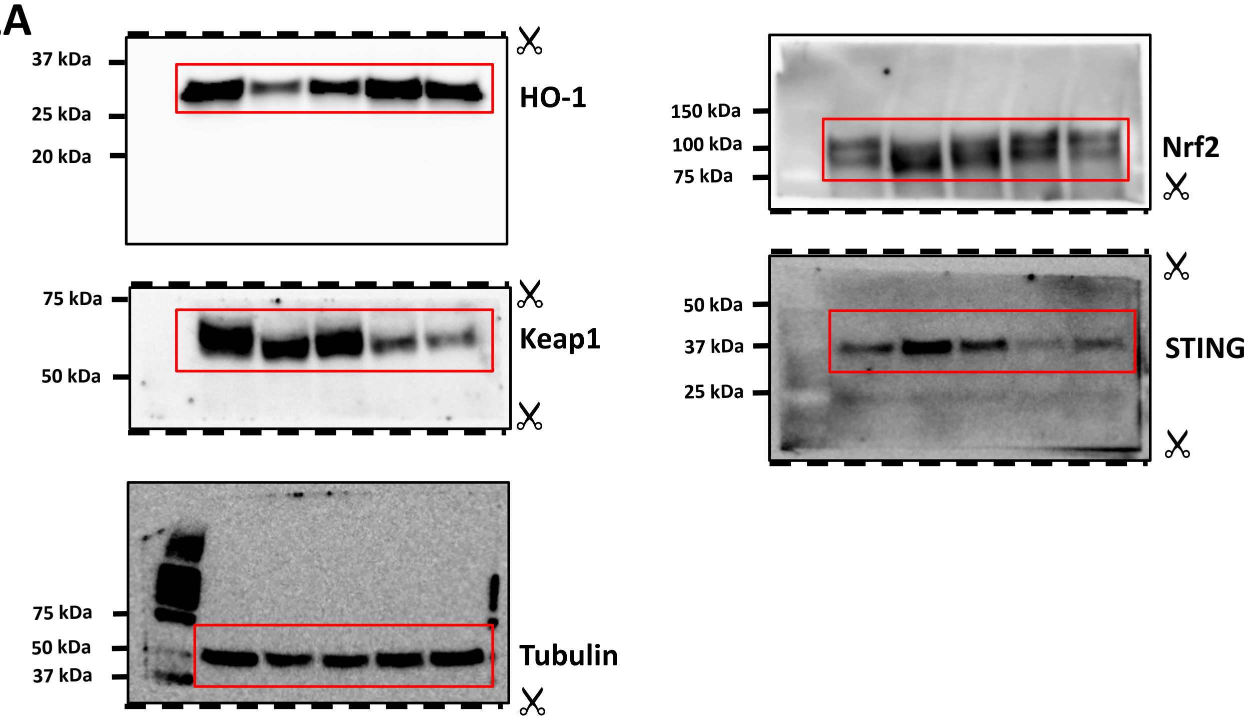

Fig. S2B

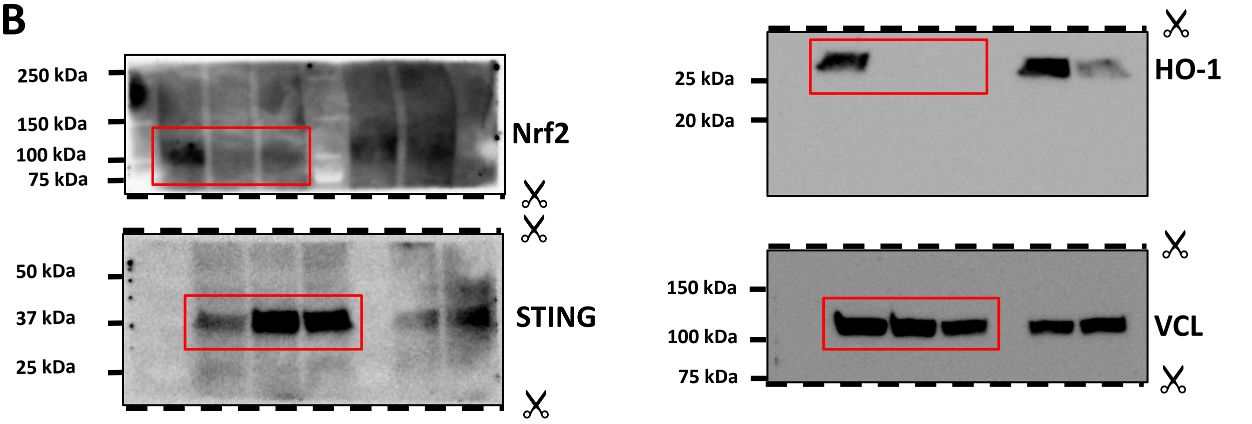

Fig. S2C

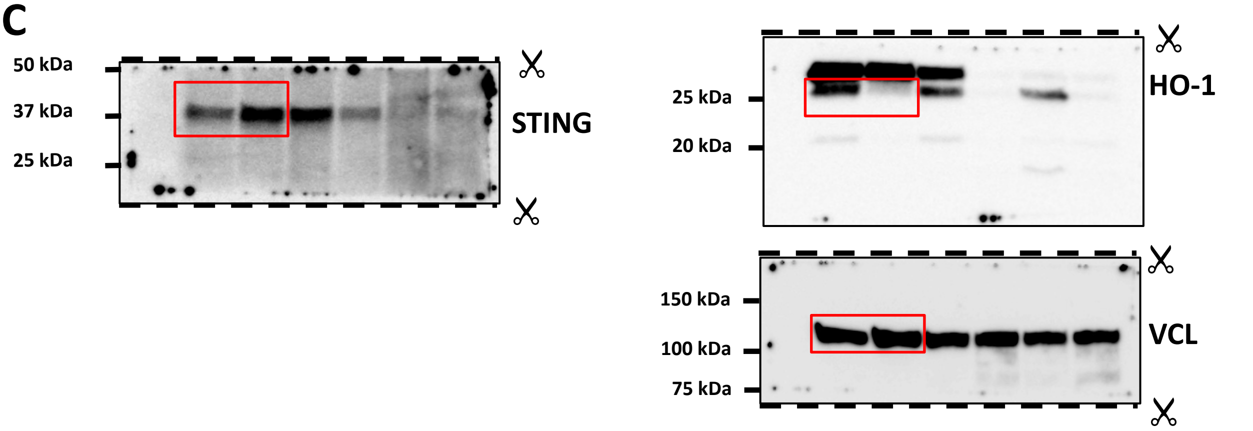

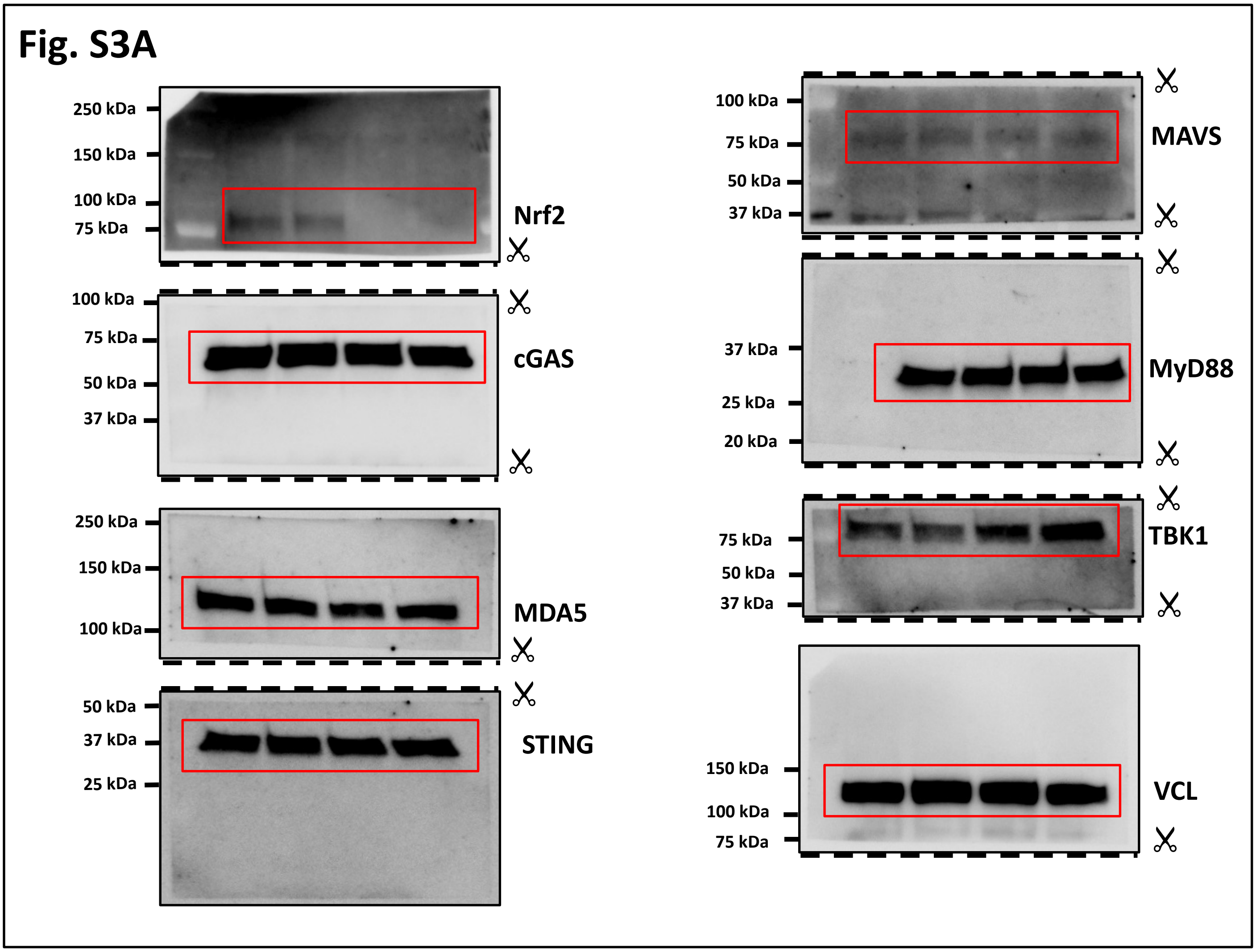
Fig. S3A
250 kDa
150 kDa
100 kDa
75 kDa
Nrf2
100 kDa
75 kDa
50 kDa
37 kDa
cGAS
250 kDa
150 kDa
100 kDa
MDA5
50 kDa
37 kDa
25 kDa
STING
100 kDa
75 kDa
50 kDa
37 kDa
MAVS
37 kDa
25 kDa
20 kDa
MyD88
75 kDa
50 kDa
37 kDa
TBK1
150 kDa
100 kDa
75 kDa
VCL

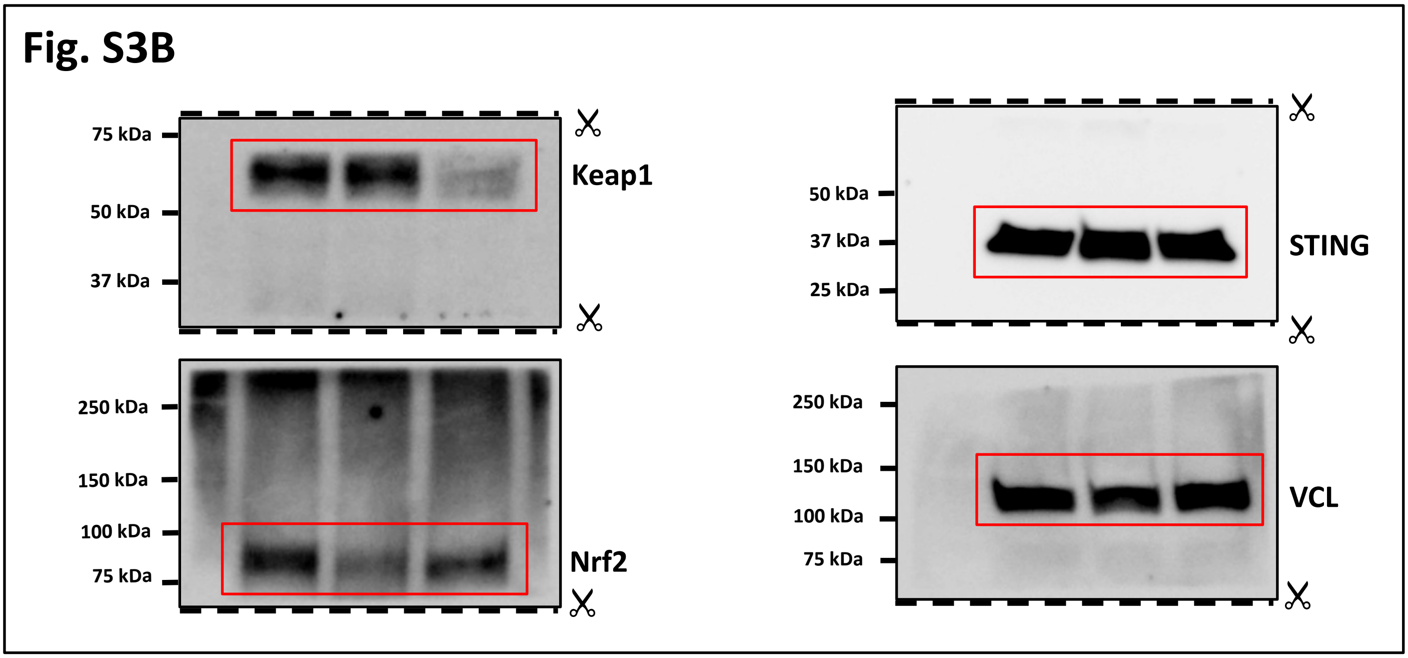
Fig. S3B
75 kDa
50 kDa
37 kDa
Keap1
250 kDa
150 kDa
100 kDa
75 kDa
Nrf2
50 kDa
37 kDa
25 kDa
STING
250 kDa
150 kDa
100 kDa
75 kDa
VCL

Fig. S5A

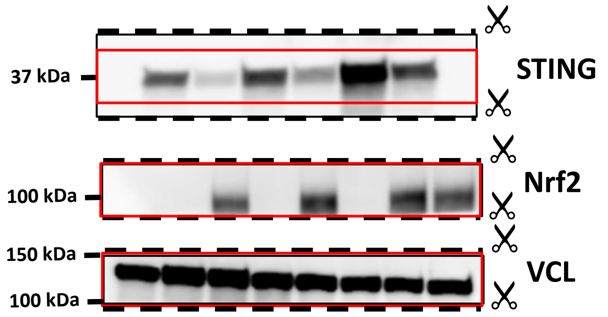

Fig. S6A

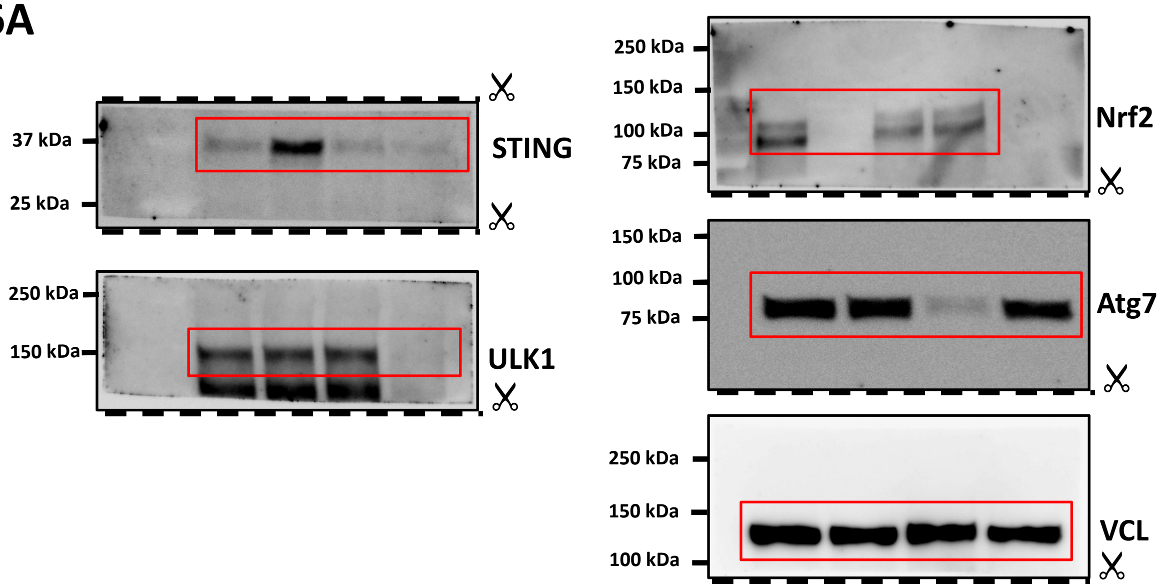

Fig. S6B

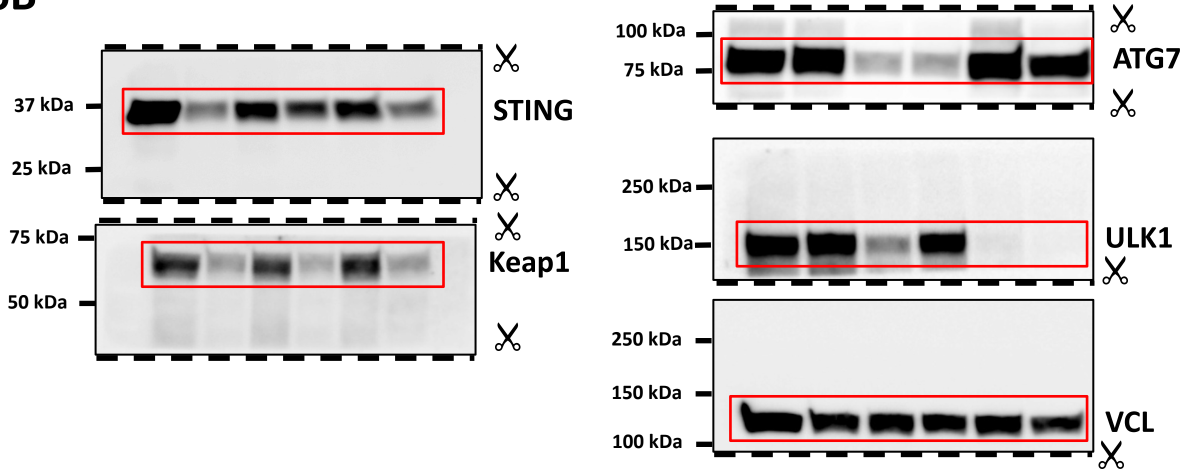

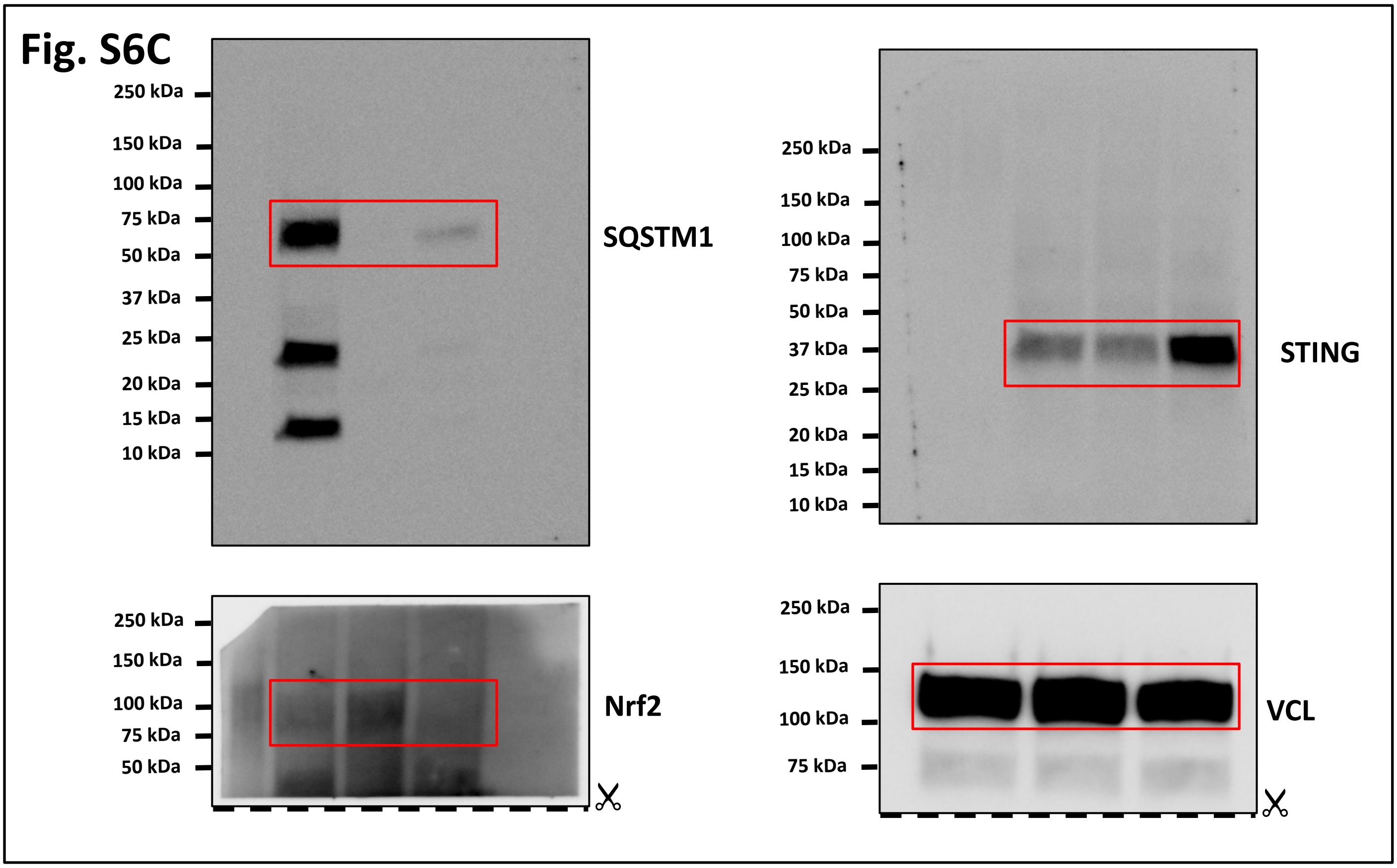

Fig. S6C
250 kDa
150 kDa
100 kDa
75 kDa
50 kDa
37 kDa
25 kDa
20 kDa
15 kDa
10 kDa
SQSTM1
STING
Nrf2
VCL

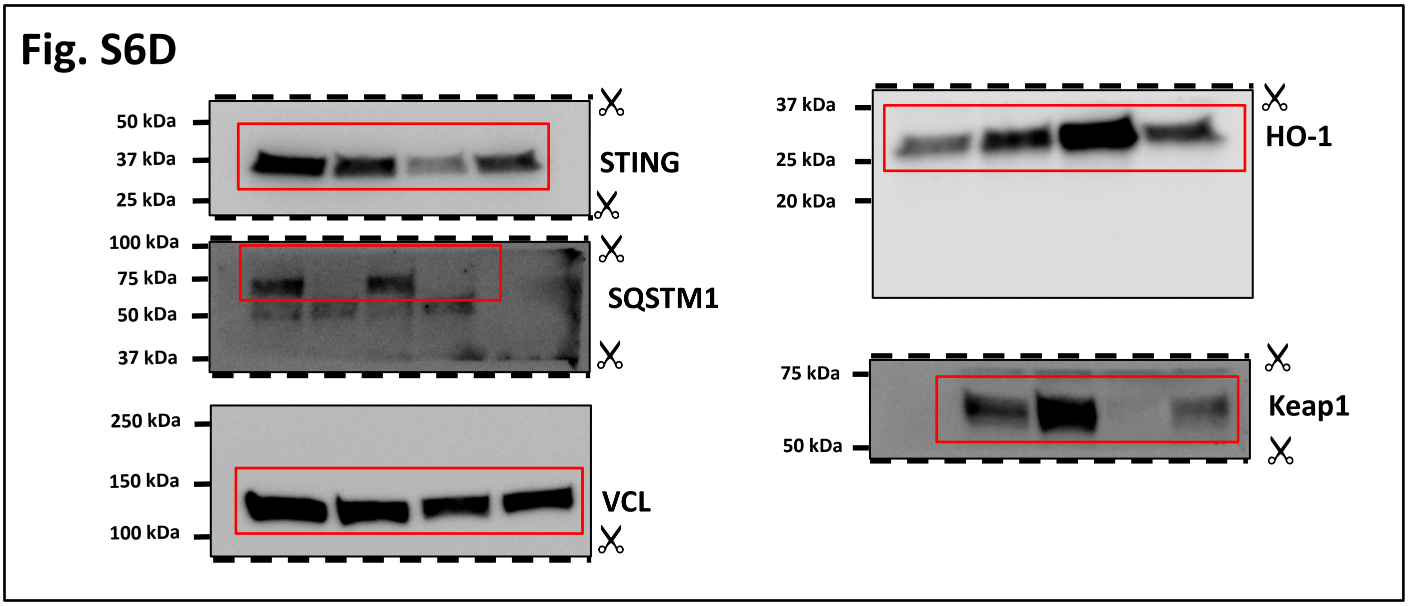

Fig. S6D
50 kDa
37 kDa
25 kDa
STING
100 kDa
75 kDa
SQSTM1
250 kDa
150 kDa
VCL
20 kDa
HO-1
Keap1

Fig. S7A

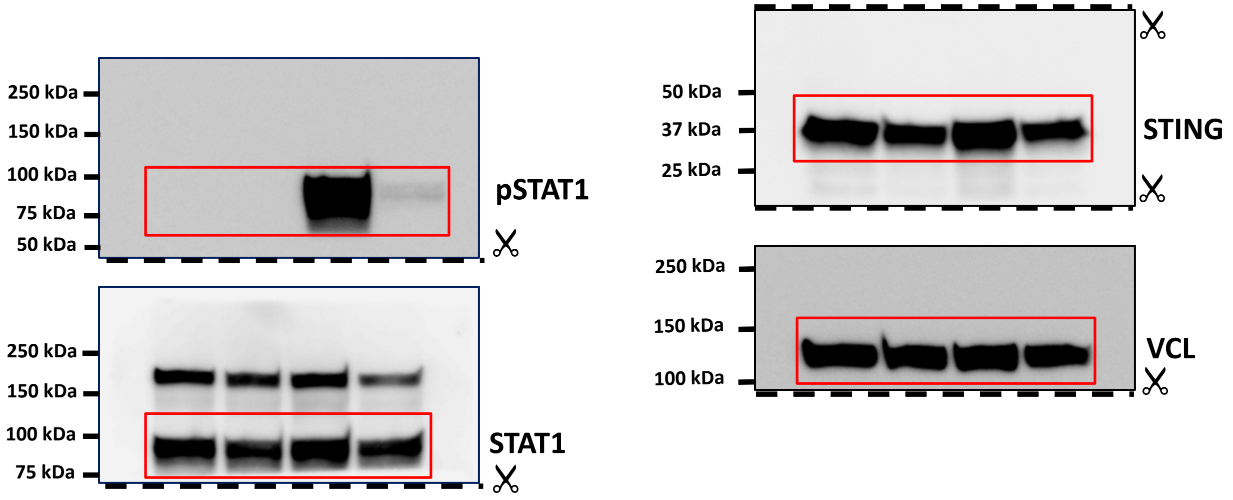

Fig. S7B

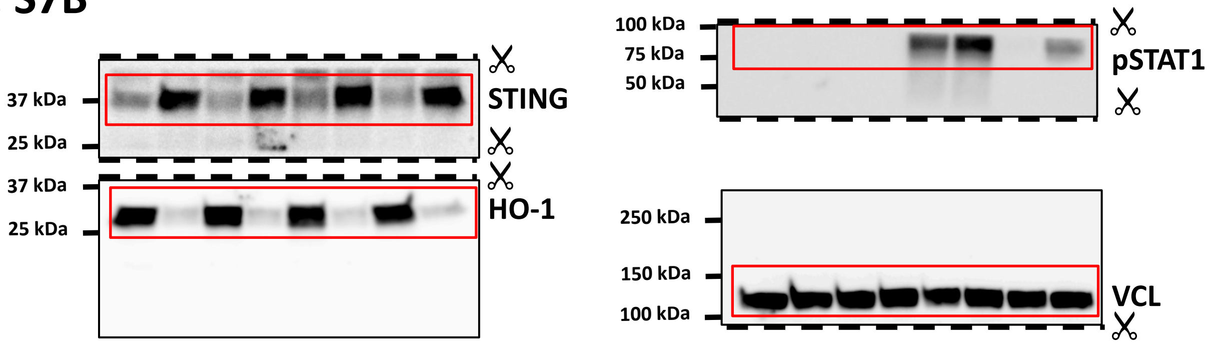

Fig. S8A

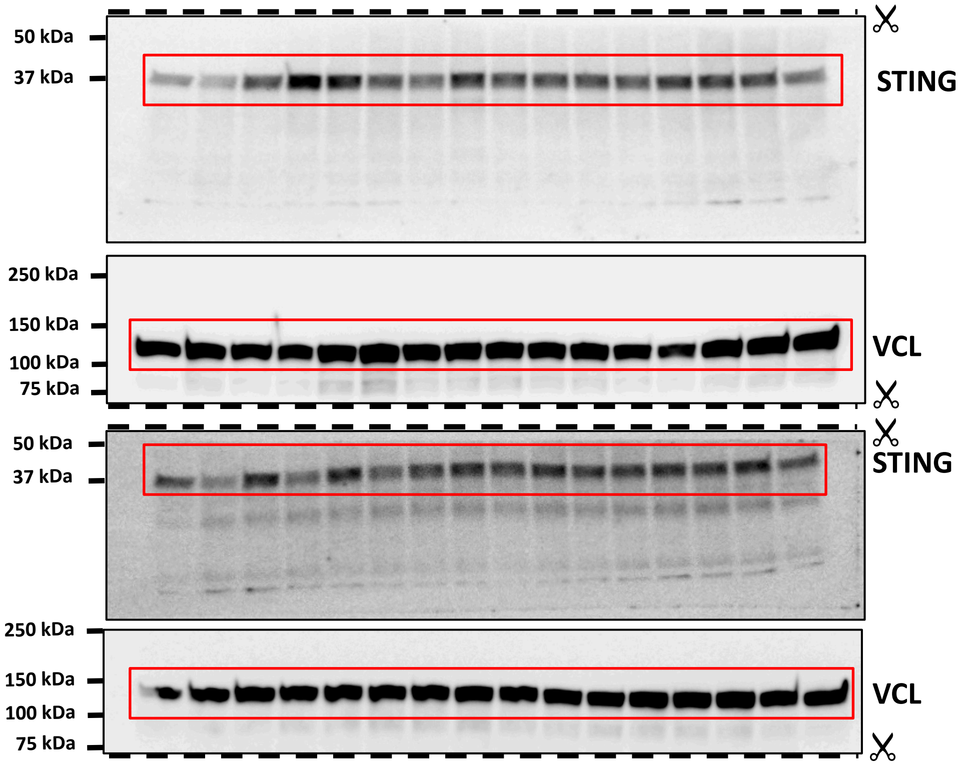

Fig. S8B

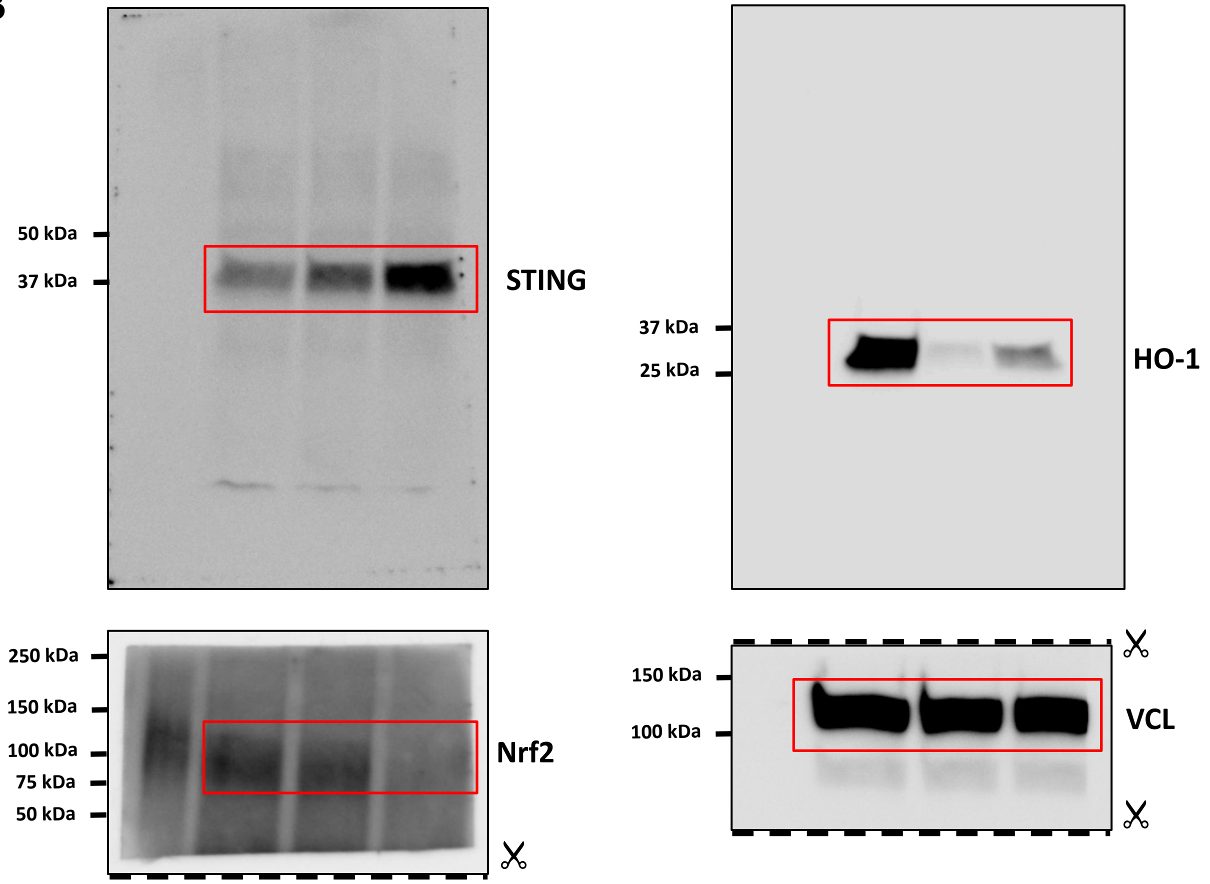

Fig. S8C

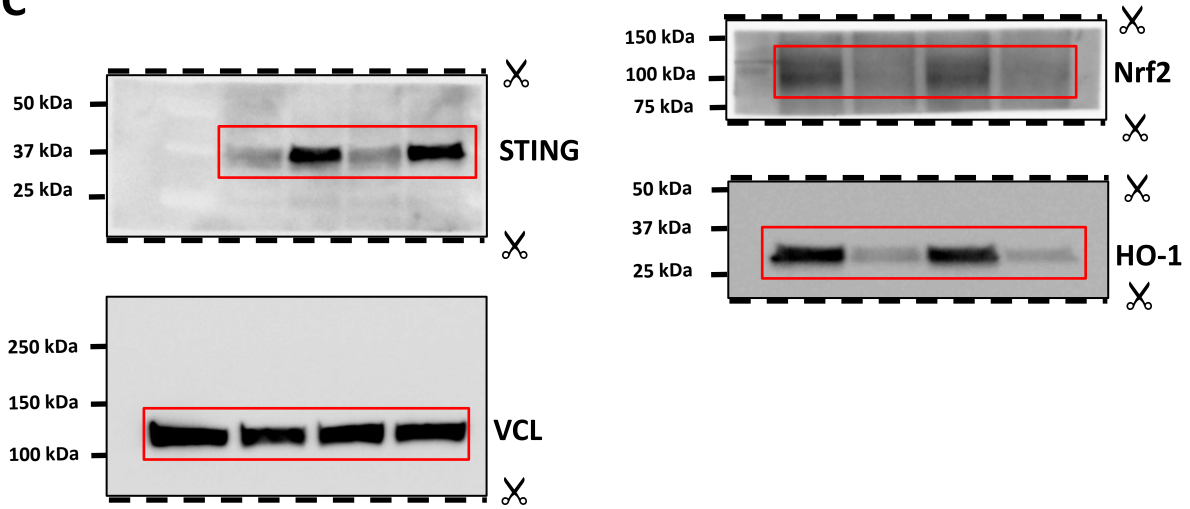

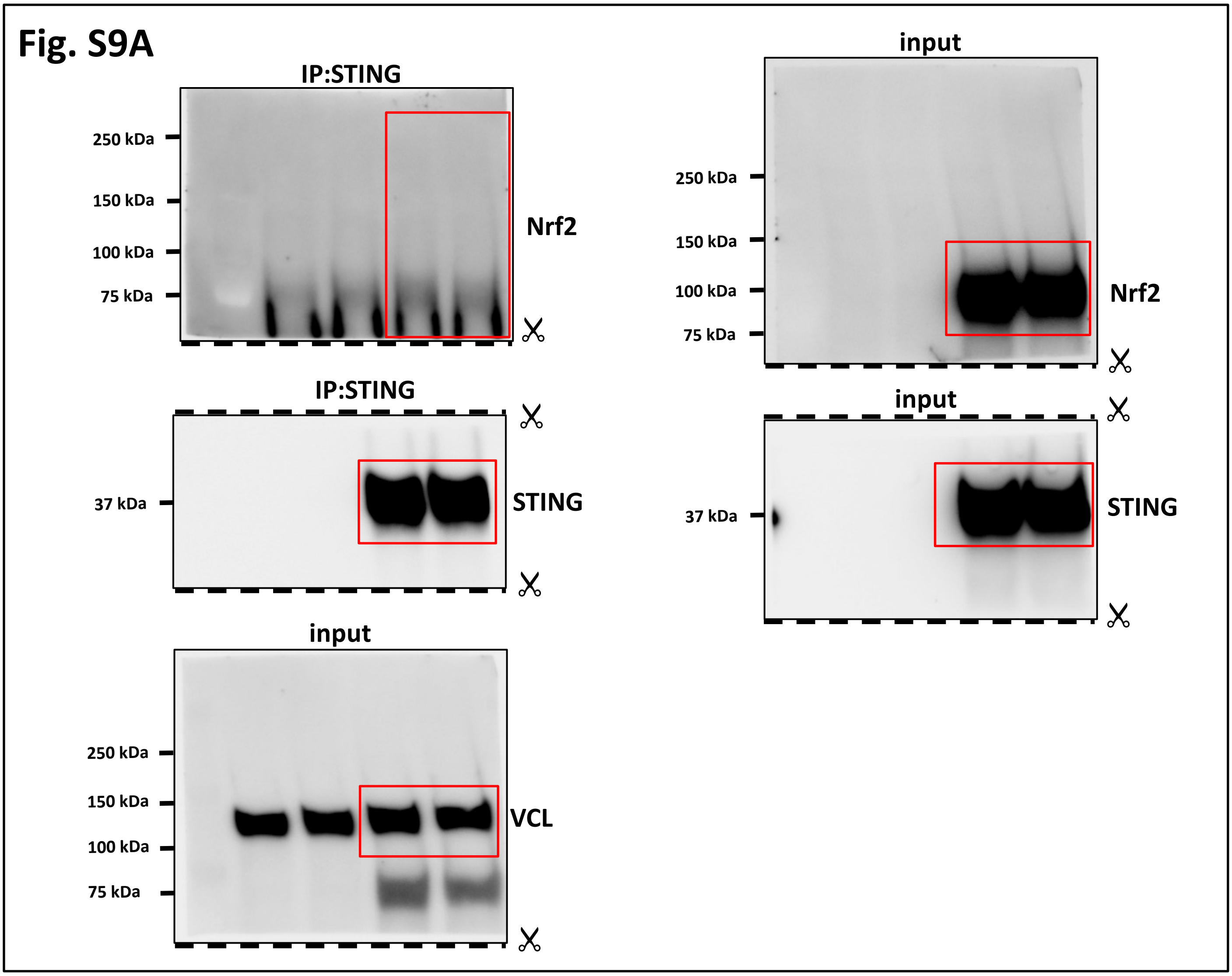

Fig. S9A
IP:STING
250 kDa
150 kDa
100 kDa
75 kDa
Nrf2
IP:STING
37 kDa
STING
input
250 kDa
150 kDa
100 kDa
75 kDa
VCL
input
250 kDa
150 kDa
100 kDa
75 kDa
Nrf2
input
37 kDa
STING

Fig. S9B

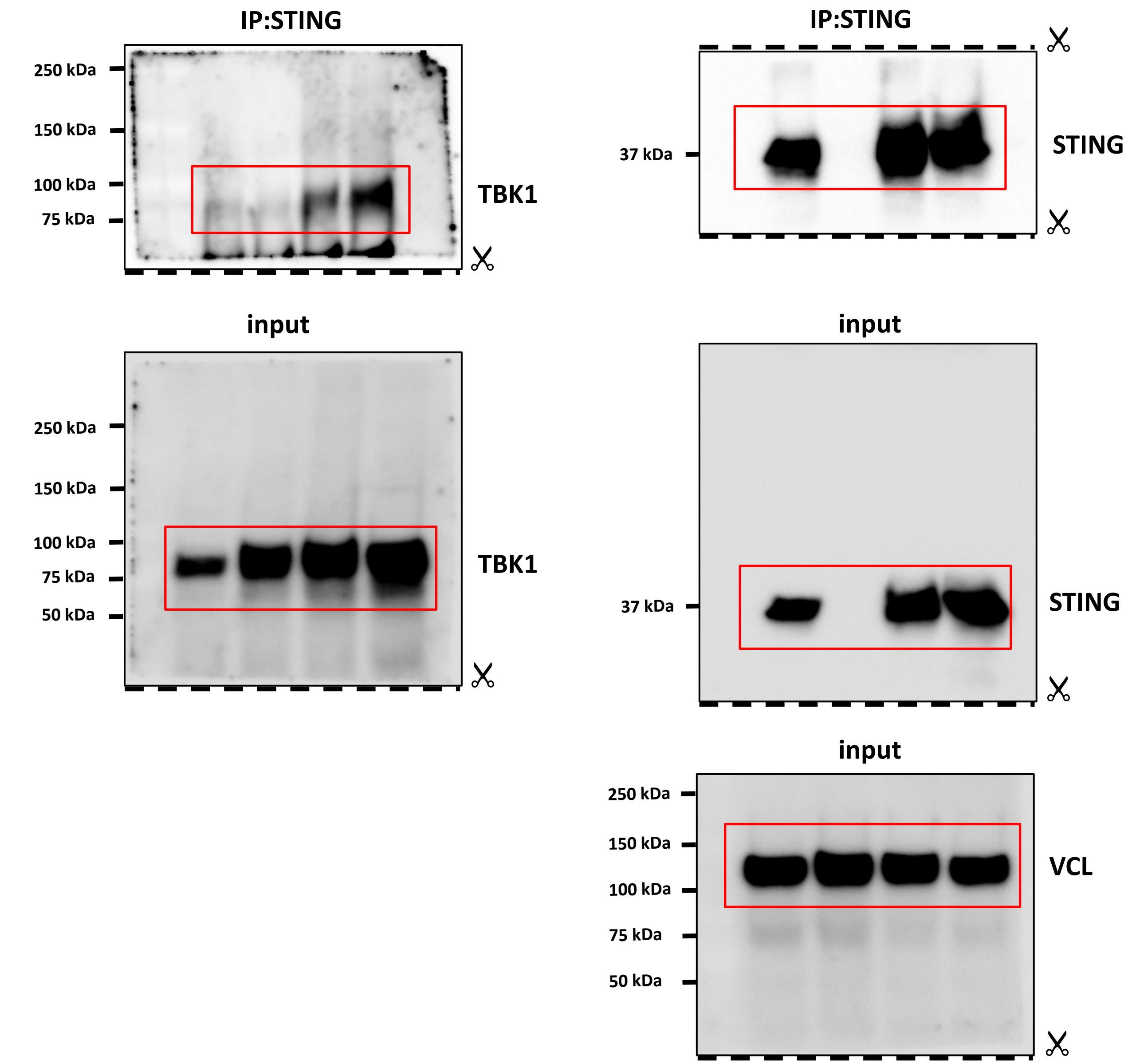
IP:STING
250 kDa
150 kDa
100 kDa
75 kDa
TBK1
IP:STING
37 kDa
STING
input
250 kDa
150 kDa
100 kDa
75 kDa
50 kDa
TBK1
input
37 kDa
STING
input
250 kDa
150 kDa
100 kDa
75 kDa
50 kDa
VCL

Fig. S9C

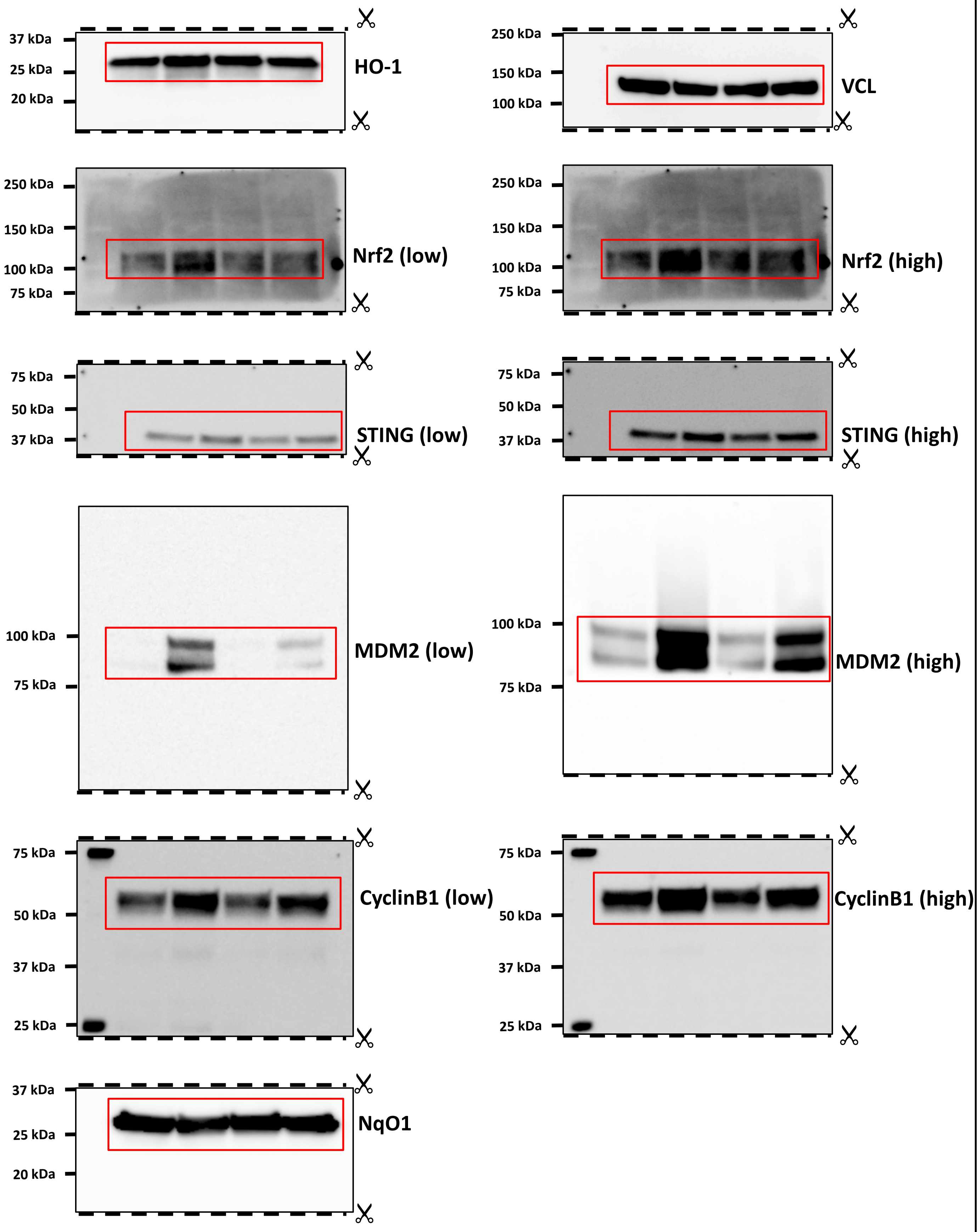
37 kDa
25 kDa
20 kDa
HO-1
250 kDa
150 kDa
100 kDa
VCL
250 kDa
150 kDa
100 kDa
75 kDa
Nrf2 (low)
250 kDa
150 kDa
100 kDa
75 kDa
Nrf2 (high)
75 kDa
50 kDa
37 kDa
STING (low)
75 kDa
50 kDa
37 kDa
STING (high)
100 kDa
75 kDa
MDM2 (low)
100 kDa
75 kDa
MDM2 (high)
75 kDa
50 kDa
37 kDa
25 kDa
CyclinB1 (low)
75 kDa
50 kDa
37 kDa
25 kDa
CyclinB1 (high)
37 kDa
25 kDa
20 kDa
NqO1

Fig. S10A

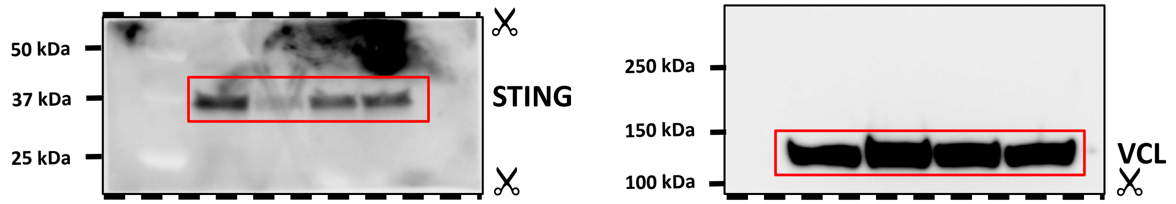

Fig. S12B

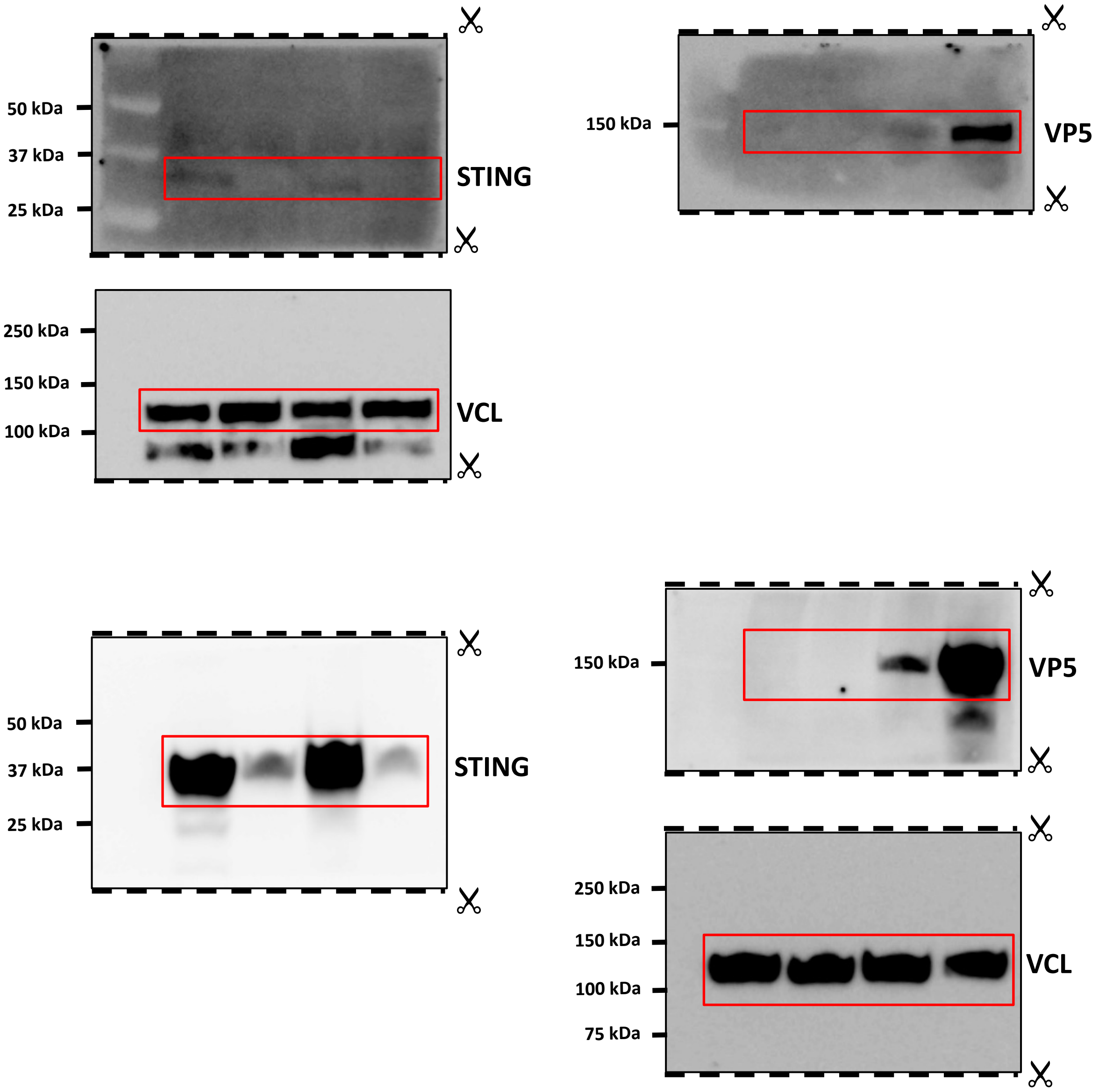

Fig. S14

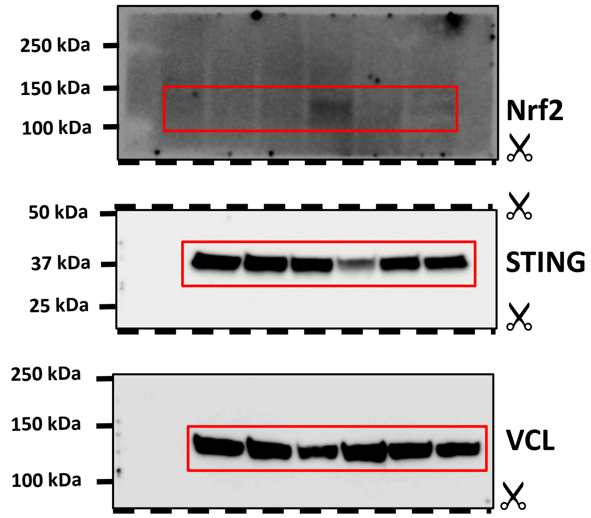
250 kDa
150 kDa
100 kDa
Nrf2
50 kDa
37 kDa
25 kDa
STING
250 kDa
150 kDa
100 kDa
VCL

Supplementary Methods

Olagnier et al.,

Organic synthesis experimental:

General:

Reagents were used as received from commercial suppliers (Sigma Aldrich, TCI, and Fluorochem). Concentration *in vacuo* was performed using a rotary evaporator with the water bath temperature at 40 °C, followed by further concentration using a high vacuum pump. TLC analysis was carried out on silica coated aluminum foil plates (Merck Kieselgel 60 F254). The TLC plates were visualized by UV irradiation and/or by staining with $KMnO_4$ stain ($KMnO_4$ (5.0 g), 5 % NaOH (aq., 8.3 mL) and K_2CO_3 (33.3 g) in H_2O (500 mL)).

Flash column chromatography (FCC) was carried out using silica gel (230-400 mesh particle size, 60 Å pore size) as stationary phase. Infrared spectra (IR) were acquired on a PerkinElmer Spectrum TwoTM UATR. Mass spectra (HRMS) were recorded on a Bruker Daltonics MicrOTOF time-of-flight spectrometer with positive electrospray ionization. Nuclear magnetic resonance (NMR) spectra were recorded on a Bruker BioSpin GmbH 400 MHz spectrometer, running at 400 and 101 MHz for ^{1}H and ^{13}C, respectively. Chemical shifts (δ) are reported in ppm relative to the residual solvent signals ($CDCl_3$ @ 7.26 ppm ^{1}H NMR, 77.16 ppm ^{13}C NMR; DMSO-d_6 @ 2.50 ppm 1H NMR, 39.52 ppm). Multiplicities are indicated using the following abbreviations: s = singlet, d = doublet, t = triplet, q = quartet, hex = hexet, m = multiplet.

1-ethyl 5-methyl 2-acetyl-2-methylpentanedioate (1)

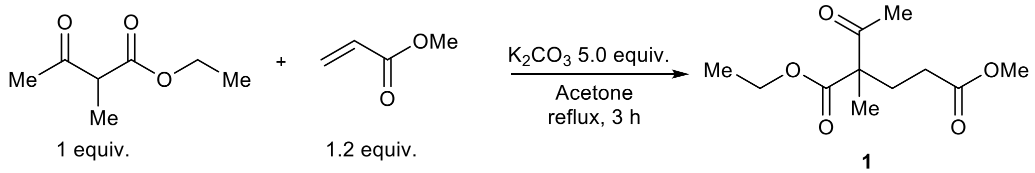

1

Ethyl 2-methyl-3-oxobutanoate (1.41 mL, 10 mmol, 1 equiv.) was dissolved in acetone (40 mL) and K_2CO_3 (6.91 g, 50 mmol, 5 equiv.) was added. Methyl acrylate (1.1 mL, 86 mmol, 1.22 equiv.) was added. A reflux condenser was equipped, and the reaction was heated to reflux while stirred vigorously. After 3 hours, the reaction was allowed to cool to ambient temperature, and the solvent was removed *in vacuo*. The crude mixture was diluted with water (60 mL) and extracted three times with Et_2O. The combined organic phases were dried over Na_2SO_4, filtered, and concentrated to yield a clear oil. The crude product from the reaction (**1**) was advanced to the next step without further purification.

4-methyl-5-oxohexanoic acid (3)

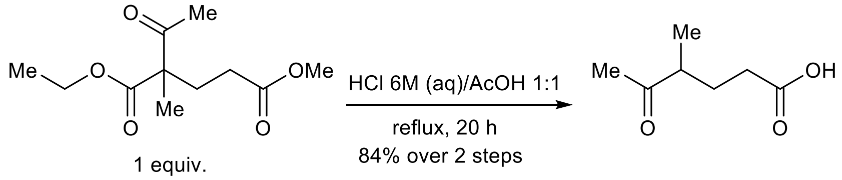

1 **2**

The crude oil **1** (10 mmol, 1 equiv.) was dissolved in neat AcOH (6 mL), and HCl (6 M, aq., 6 mL) was added to yield a clear solution. A reflux condenser was equipped, and the reaction was heated to reflux while stirred. After 20 h, the reaction was allowed to cool to ambient temperature. The reaction mixture was concentrated *in vacuo*, re-dissolved with water and extracted three times with Et_2O. The combined organic phases were dried over Na_2SO_4, filtered and concentrated. FCC (SiO_2) Et_2O/pentane 1:3 to 1:1 yielded the product **2** as a clear oil (1.21 g, 8.4 mmol, 84%). Data matches what has previously been reported.[1]

R_f	0.35 (Et_2O/pentane 1:1, $KMnO_4$); product tails.
^{1}H NMR	(400 MHz, $CDCl_3$) δ 11.58 (s, 1H), 2.62 (h, *J* = 7.0 Hz, 1H), 2.45 – 2.27 (m, 2H), 2.17 (s, 3H), 1.99 (dq, *J* = 14.8, 7.4 Hz, 1H), 1.74 – 1.58 (m, 1H), 1.12 (d, *J* = 7.1 Hz, 3H).
^{13}C NMR	(101 MHz, $CDCl_3$) δ 212.3, 178.6, 45.7, 31.3, 28.0, 26.9, 16.0.
HRMS	Calc.: $C_7H_{12}O_3Na^+$ 167.0679, found; 167.0671.
IR (neat)	vmax / cm^{-1} 2927, 1704, 1459, 1162.

ERG240

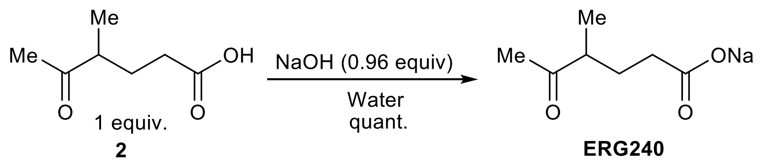

The carboxylic acid **2** (236 mg, 1.64 mmol, 1 equiv.) was dissolved in a solution of NaOH (63 mg, 1.58 mmol, 0.96 equiv.) in 2 mL water. The clear homogenous mixture was frozen with liquid N_2 and concentrated by lyophilization to yield ERG240 as white solid (272 mg, 1.64 mmol, quant). The product was soluble in D_2O and DMSO-*d_6* but not in $CDCl_3$.

^{1}H NMR	(400 MHz, DMSO-*d_6*) δ 2.07 (s, 3H), 1.81 (t, *J* = 7.3 Hz, 2H), 1.78 – 1.68 (m, 1H), 1.39 (dq, *J* = 14.1, 7.0 Hz, 1H), 0.93 (d, *J* = 6.9 Hz, 3H).

2-methylene-4-(octyloxy)-4-oxobutanoic acid (3)

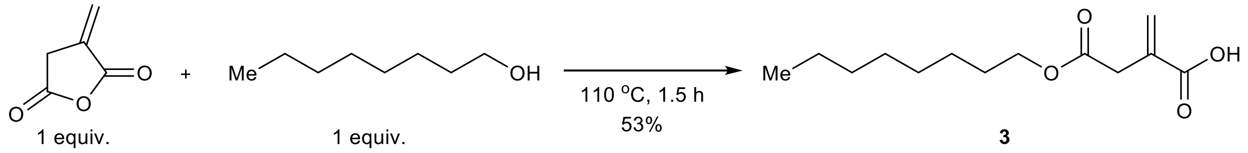

1-Octanol (5.62 mL, 35.7 mmol, 1 equiv.) was heated to 110 °C in a flask. 3-methylene-dihydrofuran-2,5-dione (4.0 g, 35.7 mmol, 1 equiv.) was added, everything dissolved yielding a clear solution. After 90 min, the mixture was poured into 100 mL heptane and stirred at ambient temperature. The mixture starts as a clear solution, slowly a white solid is formed. After 3 hours, the white solid product **3** was filtered off, washed with heptane and dried under vacuum (4.6 g, 19 mmol, 53%).

R_f	0.22 (EtOAc/heptane 4:6, $KMnO_4$); product tails.
^{1}H NMR	(400 MHz, $CDCl_3$) δ 6.46 (d, *J* = 0.9 Hz, 1H), 5.83 (q, *J* = 1.1 Hz, 1H), 4.10 (t, *J* = 6.7 Hz, 2H), 3.34 (d, *J* = 1.0 Hz, 2H), 1.67 – 1.56 (m, 2H), 1.38 – 1.19 (m, 10H), 0.88 (t, *J*= 6.7 Hz, 3H).
^{13}C NMR	(101 MHz, $CDCl_3$) 170.9, 170.8, 133.3, 130.8, 65.4, 37.5, 31.9, 29.3, 29.3, 28.6, 26.0, 22.8, 14.3.
HRMS	Calc.: $C_{13}H_{22}O_4Na^+$ 265.1210; found 265.1209
IR (neat)	vmax / cm^{-1} 2953, 2917, 2851, 1722, 1683, 1634, 1185, 1168.

Spectra:

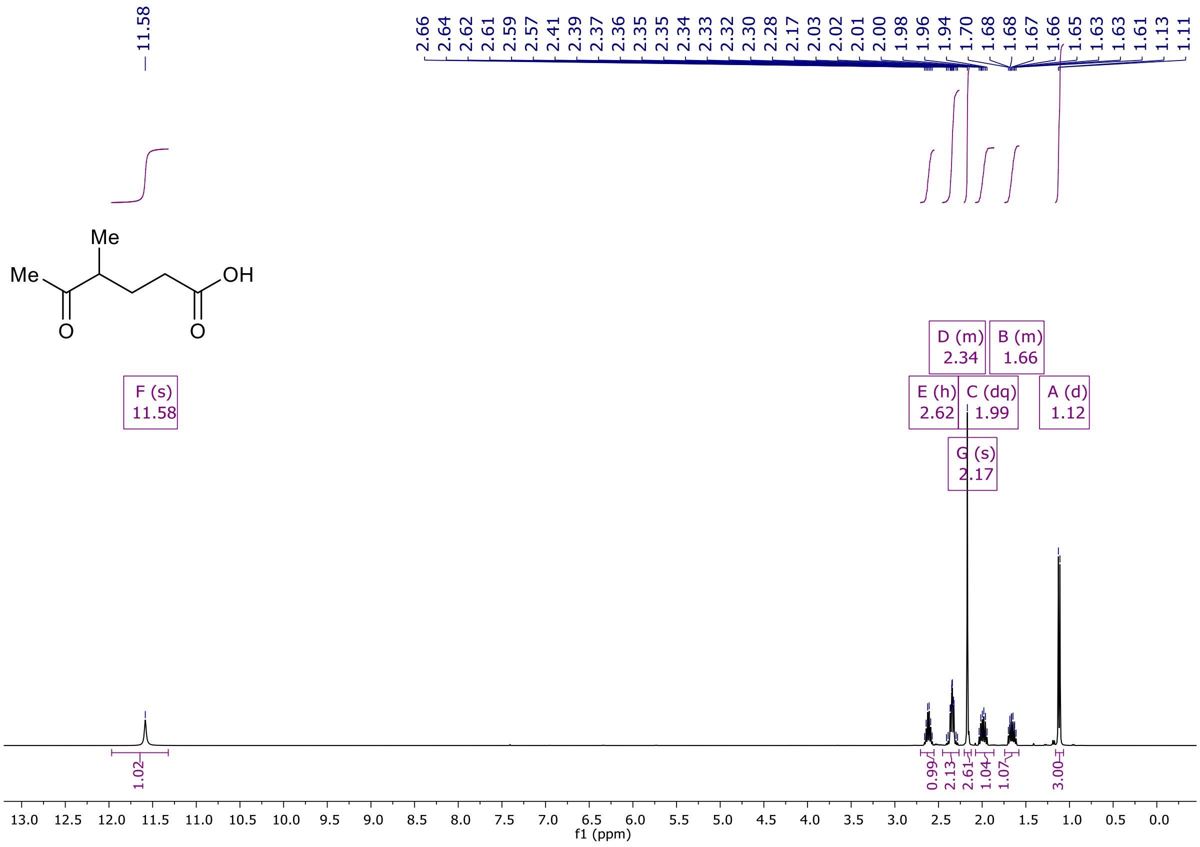

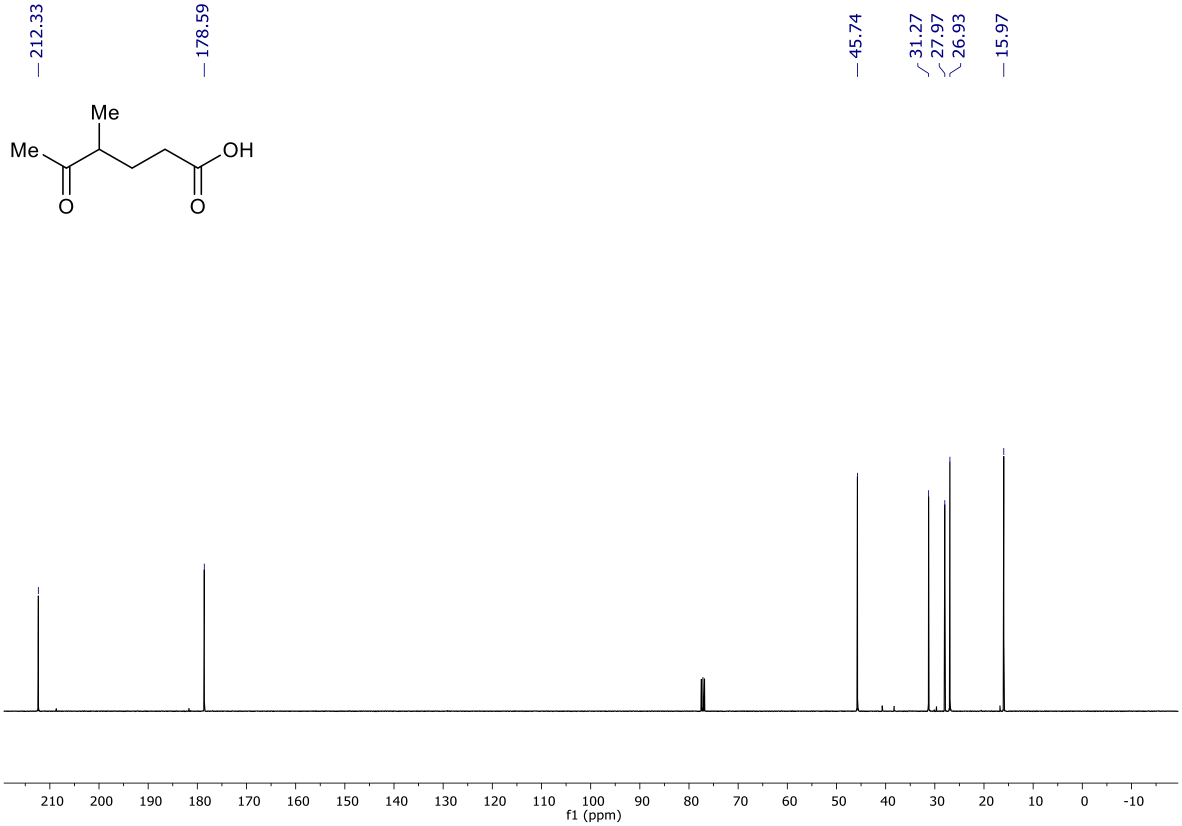

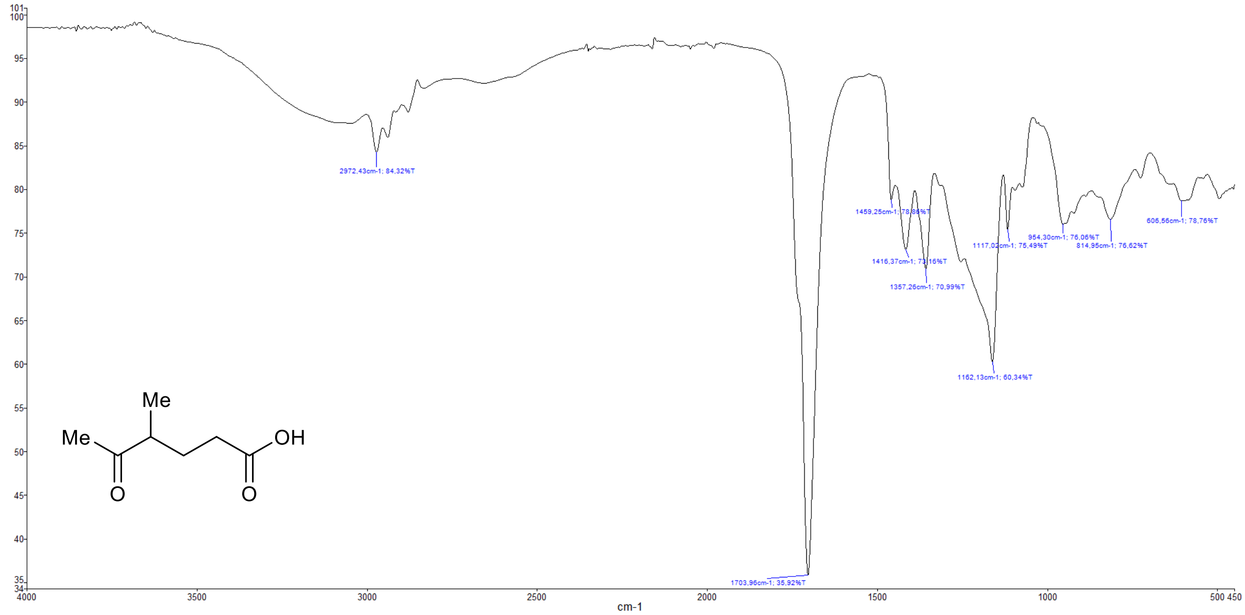
Me
Me
OH
O
O
2972,43cm-1; 84,32%T
1357,26cm-1; 70,99%T
1117,02cm-1; 75,49%T
954,30cm-1; 76,06%T
814,95cm-1; 76,62%T
606,56cm-1; 78,76%T
1162,13cm-1; 60,34%T
1703,96cm-1; 35,92%T
cm-1

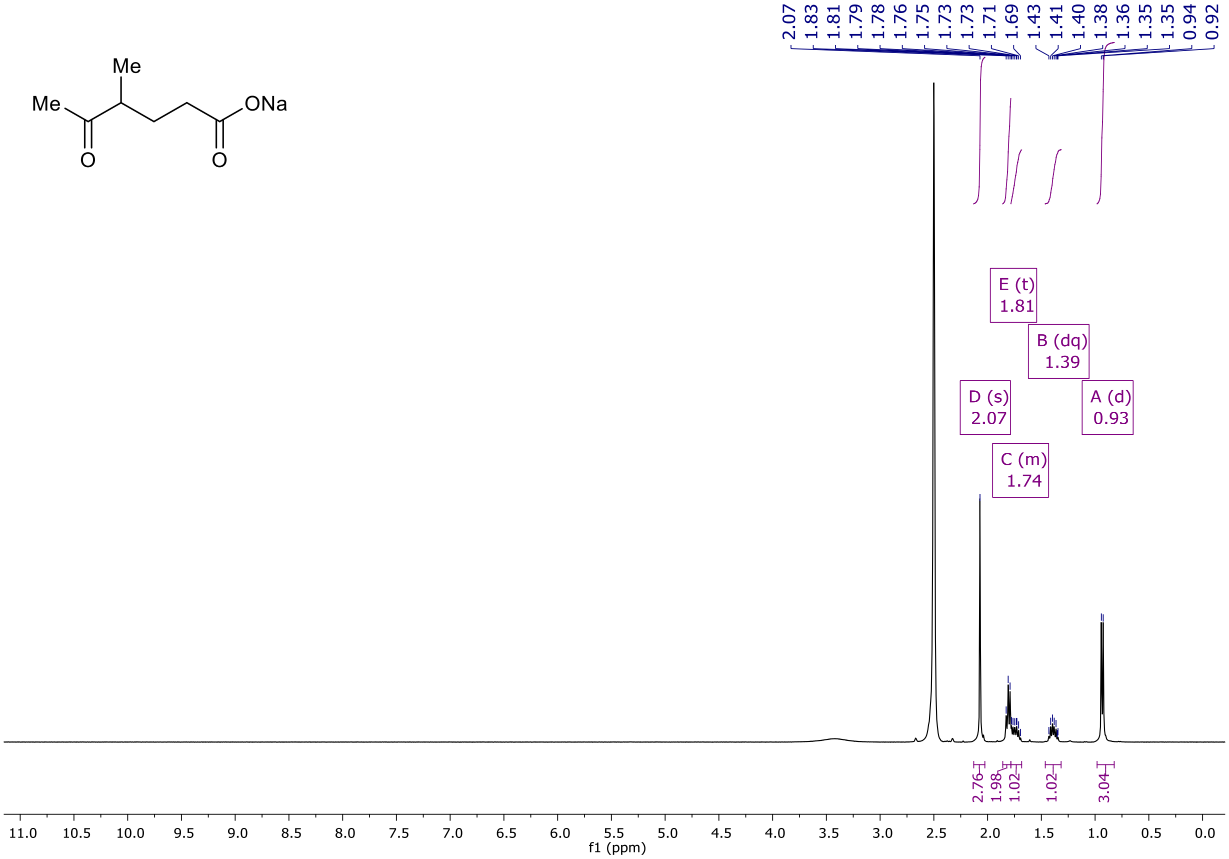
Me
Me
ONa
O
O
2.07 1.83 1.81 1.79 1.78 1.76 1.75 1.73 1.73 1.71 1.69 1.43 1.41 1.40 1.38 1.36 1.35 1.35 0.94 0.92
E (t) 1.81
B (dq) 1.39
D (s) 2.07
A (d) 0.93
C (m) 1.74
2.76
1.98
1.02
1.02
3.04
f1 (ppm)

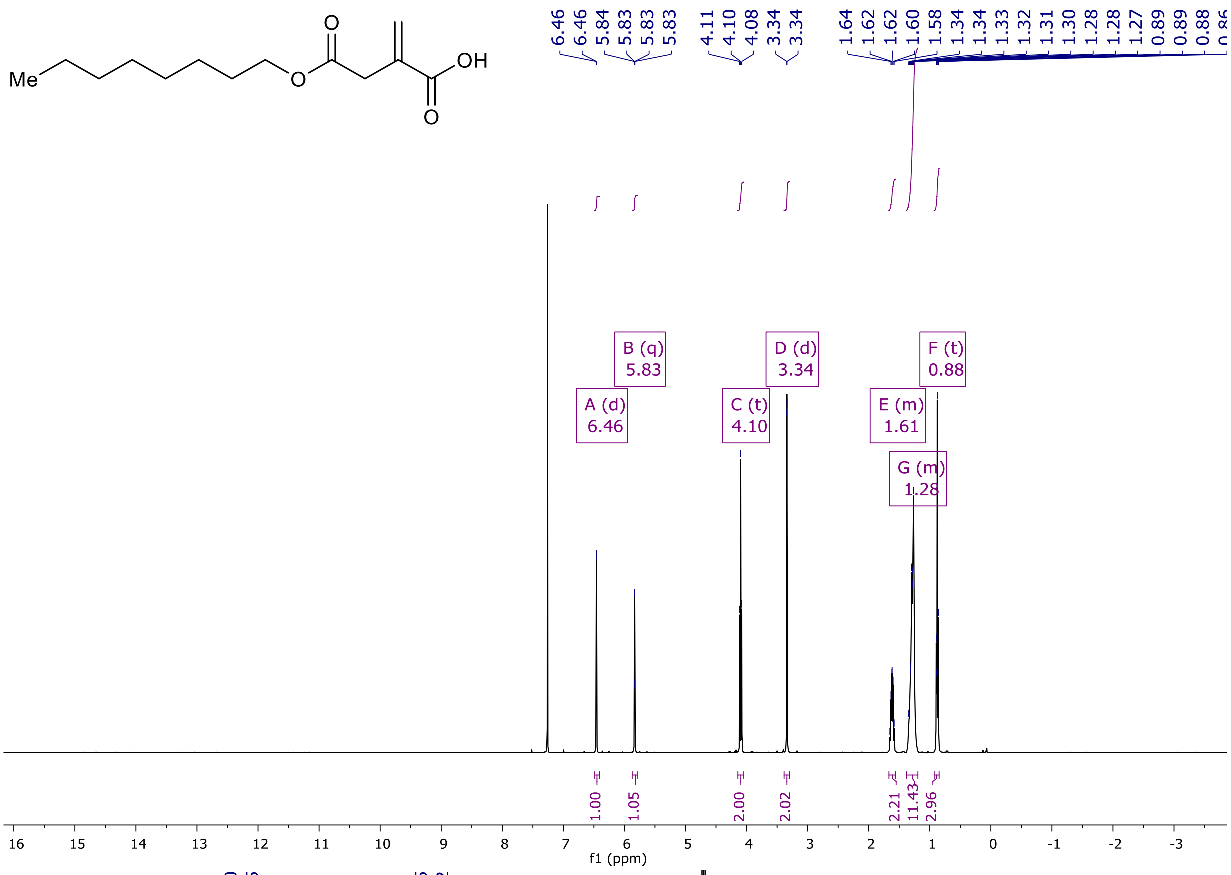

Me
O
OH
O
6.46
6.46
5.84
5.83
5.83
5.83
5.83
4.11
4.10
4.08
3.34
3.34
1.64
1.62
1.62
1.60
1.58
1.34
1.34
1.33
1.32
1.31
1.30
1.28
1.28
1.27
0.89
0.89
0.88
A (d) 6.46
B (q) 5.83
C (t) 4.10
D (d) 3.34
E (m) 1.61
G (m) 1.28
F (t) 0.88
1.00
1.05
2.00
2.02
2.21
11.43
2.96
16 15 14 13 12 11 10 9 8 7 6 5 4 3 2 1 0 -1 -2 -3
f1 (ppm)

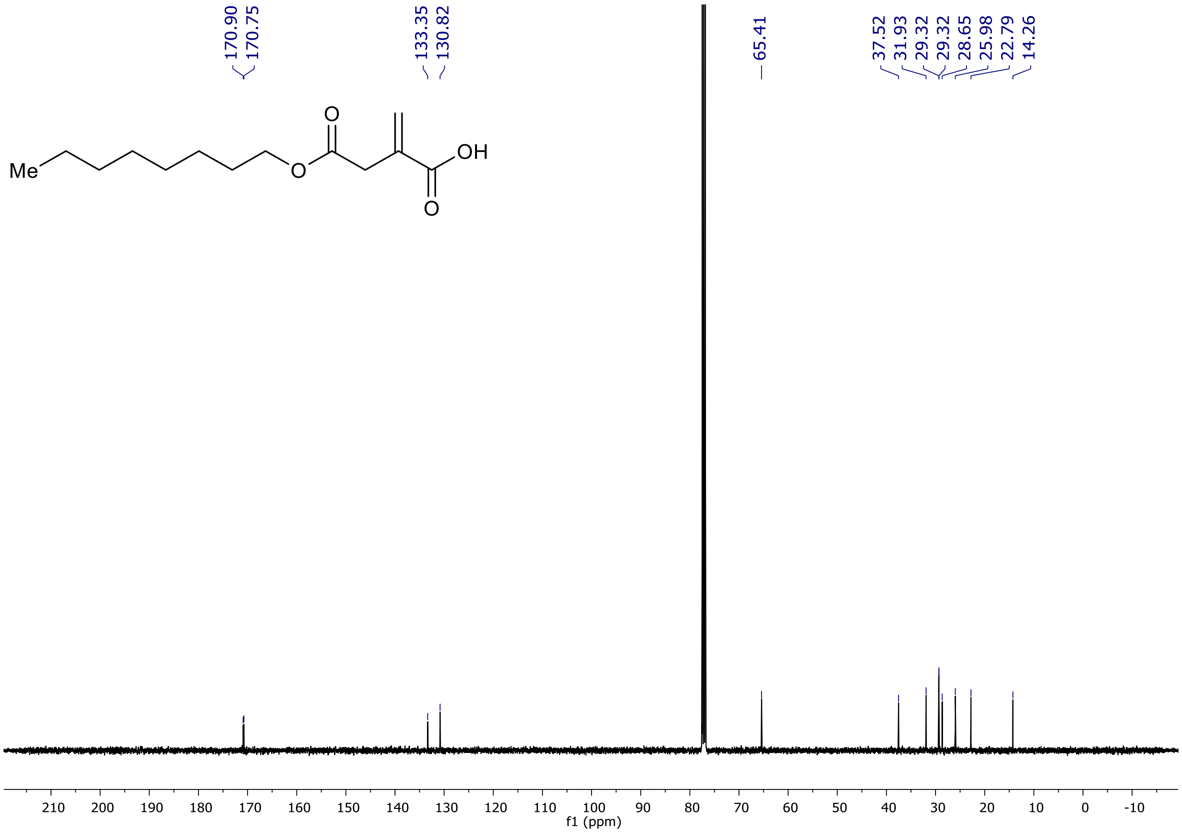

170.90
170.75
133.35
130.82
65.41
37.52
31.93
29.32
29.32
28.65
25.98
22.79
14.26
Me
O
OH
O
210 200 190 180 170 160 150 140 130 120 110 100 90 80 70 60 50 40 30 20 10 0 -10
f1 (ppm)

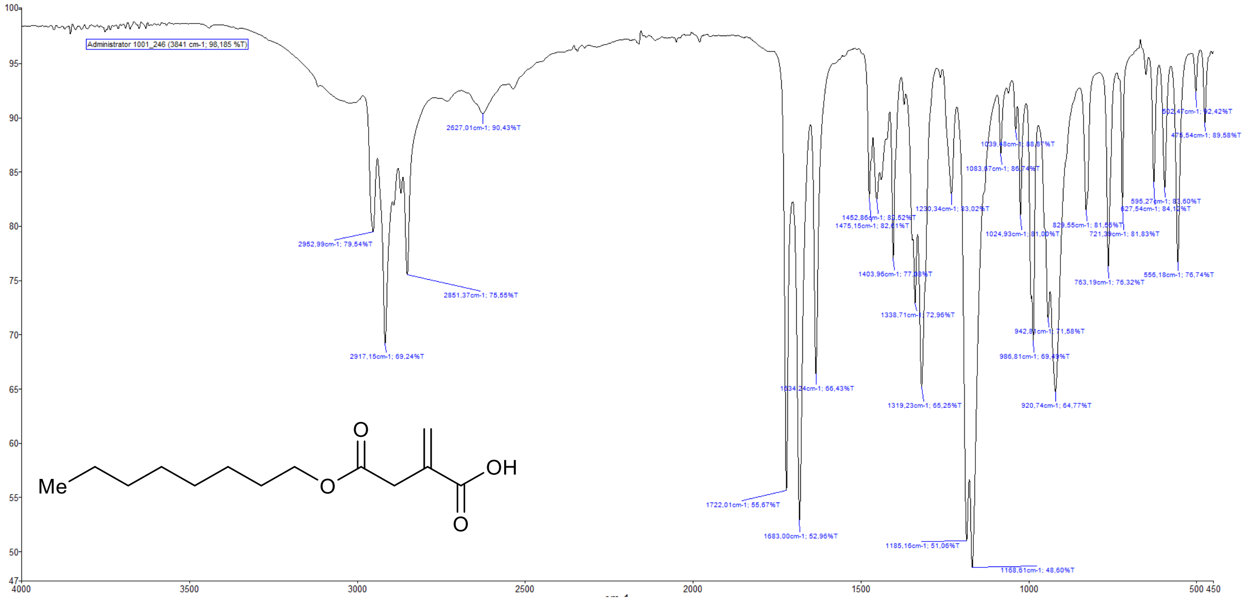

References:

1: Holloway, C. A., Muratore, M. E., Storer, R. I. & Dixon, D. J. Direct Enantioselective Brønsted Acid Catalyzed *N*-Acyliminium Cyclization Cascades of Tryptamines and Ketoacids. Org. Lett. 12, 4720-2723 (2010)
